# An amphiregulin reporter mouse enables transcriptional and clonal expansion analysis of reparative lung Tregs

**DOI:** 10.1172/jci.insight.187245

**Published:** 2025-07-08

**Authors:** Lucas F. Loffredo, Katherine A. Kaiser, Adam Kornberg, Samhita Rao, Kenia de los Santos-Alexis, Arnold Han, Nicholas Arpaia

**Affiliations:** 1 Department of Microbiology & Immunology;; 2 Columbia Center for Translational Immunology;; 3 Division of Digestive and Liver Diseases, Department of Medicine; and; 4 Herbert Irving Comprehensive Cancer Center, Columbia University, New York, New York, USA.

**Keywords:** Cell biology, Immunology, Influenza

## Abstract

Regulatory T cells (Tregs) are known to play critical roles in tissue repair via provision of growth factors, such as amphiregulin (Areg). Areg-producing Tregs have previously been difficult to study because of an inability to isolate live Areg-producing cells. In this report, we created a reporter mouse to detect Areg expression in live cells (*Areg^Thy1.1^*). We employed influenza A and bleomycin models of lung damage to sort Areg-producing and non-Areg-producing Tregs for transcriptomic analyses. Single-cell RNA-Seq revealed distinct subpopulations of Tregs and allowed transcriptomic comparisons of damage-induced populations. Single-cell TCR sequencing showed that Treg clonal expansion was biased toward Areg-producing Tregs and largely occurred within damage-induced subgroups. Gene module analysis revealed functional divergence of Tregs into immunosuppression-oriented and tissue repair–oriented groups, leading to identification of candidate receptors for induction of repair activity in Tregs. We tested these using an ex vivo assay for Treg-mediated tissue repair, identifying 4-1BB agonism as a mechanism for reparative activity induction. Overall, we demonstrate that the *Areg^Thy1.1^* mouse is a promising tool for investigating tissue repair activity in leukocytes.

## Abstract 2

Treg cells, beyond their immunosuppressive functions, have the ability to repair damaged tissue. We developed a reporter mouse to interrogate reparative subsets of these cells.

## Introduction

The immune response in damaged tissue must be tightly coordinated to effectively clear pathogens while allowing for proper resolution and repair. For the latter to occur correctly, immune activity must be dampened following pathogen removal to prevent further damage by the immune system itself. Regulatory T cells (Tregs) are chiefly known for their roles as critical mediators of this immunosuppression (1); however, recent studies have identified additional roles for Tregs in tissue repair — as sources of direct signals to nonhematopoietic cells that regulate reparative processes. Several Treg-derived factors targeting different types of tissue cells have been identified to this effect (2), with the most studied being the EGFR ligand family growth factor amphiregulin (Areg). The reparative effects of Areg production by Tregs have been shown in multiple damaged tissue environments, including muscle (3), brain (4), heart (5), and lung (6).

Since the discovery of the pro-repair function of Tregs, several questions have arisen as to the nature of Tregs serving in this role. Although studies have pointed to a context-dependent induction of reparative functionality induced by alarmins such as IL-33 or inflammatory mediators such as IL-18 (6), whether reparative Tregs represent a stable ontogenetically separate subtype from immunosuppression-oriented Tregs is still unknown. Further, although Treg clones found to be expanded in adipose tissue and damaged muscle have been characterized for their tissue homing and repair capacity (7, 8), it has yet to be definitively determined if reparative Tregs derive from uniquely clonally expanded Tregs in response to T cell receptor (TCR) activation in damaged tissues. Importantly, the identification of pathways capable of inducing the pro-repair phenotype of Tregs could be therapeutically leveraged to limit tissue damage in various sterile and pathogenic contexts.

Separate from these lines of investigation, there is a more general knowledge gap regarding T cell clonal expansion in response to nonpathogenic, sterile organ damage. While clonal expansion/effector activity toward pathogenic agents in tissue is at the core of our understanding of the adaptive immune response, this paradigm does not address the activity of T cells in damaged tissue in the absence of a pathogen. In many of these scenarios, T cells are recruited to and rapidly proliferate at the site of damage (9). This includes Tregs, which are greatly expanded in response to nonpathogenic damage in muscle, brain, heart, skin, and liver (3–5, 10, 11). Although expansion of Tregs in the lung has mostly been studied in the context of pathogenic damage (e.g., influenza virus) or scenarios mimicking pathogen exposure (e.g., LPS), this increased prevalence has also been shown to occur in models of sterile lung damage, such as bleomycin exposure (12). Whether Tregs in lung tissue undergoing sterile damage are clonally expanded, and whether this relates to their tissue-reparative functions, is currently unclear.

The capacity to answer these questions regarding reparative Tregs has been hindered by the inability to isolate live, repair-oriented Tregs in a laboratory environment for downstream analyses. To address this deficiency, we generated a mouse strain that identifies Areg-producing Tregs. Subsequently, we used this reporter strain to isolate reparative Tregs in models of lung disease/damage for RNA and TCR sequencing. We utilized these datasets to identify pathways relevant to Treg repair functions, characterize diseased-induced subsets of lung tissue Tregs, and explore clonal expansion of Tregs in response to sterile lung damage. Finally, we applied the findings from our sequencing studies to an in vitro assay of Treg-mediated repair, uncovering 4-1BB as an inducer of reparative activity.

## Results

### The Areg^Thy1.1^ reporter mouse delineates active Areg production from Tregs during models of lung damage.

**Figure 1:**
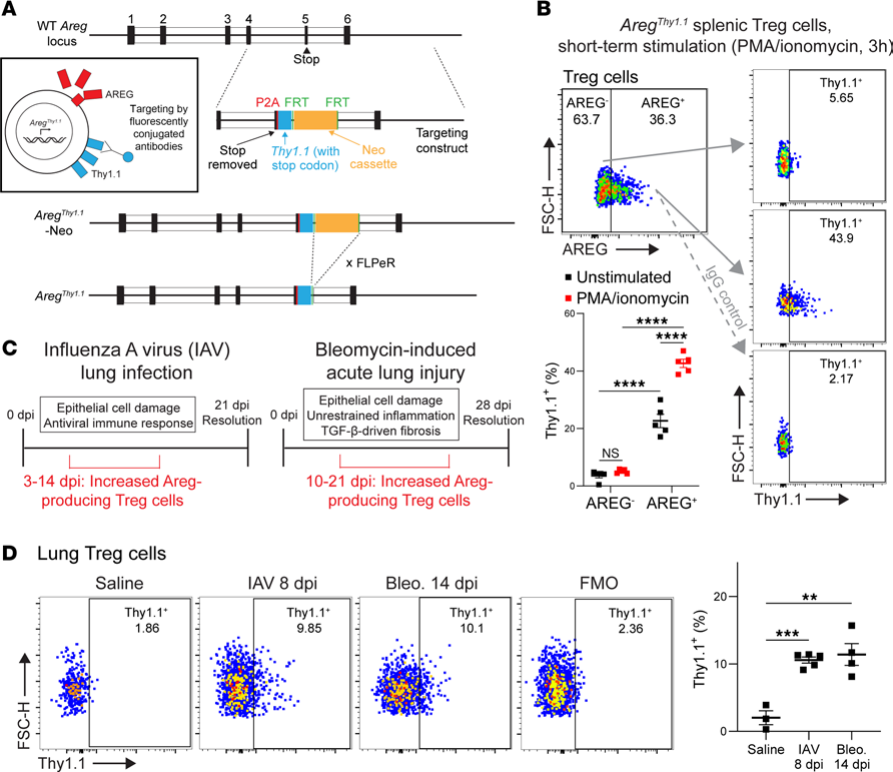
The *Areg^Thy1.1^* reporter mouse delineates active Areg production from Tregs during models of lung damage. (**A**) Schematic depicting genetic targeting of the endogenous *Areg* locus via homologous recombination with the P2A-*Thy1.1*-STOP-Neomycin knockin construct, with subsequent crossing to the FLPeR mouse to remove the neomycin cassette and create the final *Areg^Thy1.1^* allele. Inset: Depiction of Areg transcription/translation in the *Areg^Thy1.1^* mouse; for each molecule of *Areg* mRNA translated, a single *Thy1.1* mRNA is also translated (as a separate protein, due to the P2A site) and traffics separately to the surface of the cell, where it can be targeted by fluorescently conjugated antibodies. (**B**) Mouse splenocytes from *Areg^Thy1.1^* mice underwent a short-term stimulation protocol (PMA/ionomycin, 3 hours), then were stained for endogenous AREG (see Methods) and Thy1.1 (live staining). Representative plots for stimulated Tregs from AREG^–^ and AREG^+^ populations shown (including IgG control for the AREG^+^ population); plots for unstimulated Tregs not shown. Percentage staining shown on plots. Gating strategy shown in Supplemental Figure 2. *n* = 5 per group, all values included from 2 experiments. (**C**) Schematics depicting the models of lung damage used in this study, including general time course and disease characteristics, and Treg increases/Areg production status. dpi, days post-instillation. (**D**) Thy1.1 staining by flow cytometry on live lung Tregs from *Foxp3^GFP^* *Areg^Thy1.1^* mice during the IAV or bleomycin (bleo.) models of lung damage or from control (saline-treated) lungs. Dpi for each model indicated in figure. Representative plots shown, including fluorescence-minus-one (FMO) control (from IAV 8 dpi staining). Gating strategy shown in Supplemental Figure 3. *n* = 3–5 per group, all values included from 2 experiments. Mean ± SEM displayed on graphs. Statistical analysis was done using Bonferroni’s multiple comparisons test. **: 0.001 < *P* < 0.01, ***: 0.0001 < *P* < 0.001, ****: *P* < 0.0001.

Due to the inability to stain for Areg on the cell surface, isolation of live Areg-producing Tregs has not been achievable. To create a way to isolate these cells, we generated a murine reporter system: the *Areg^Thy1.1^* knockin mouse. This was done by insertion of a sequence encoding a self-cleaving *Thy1^a^* (*Thy1.1*) construct into the endogenous *Areg* locus, wherein whenever a transcript of *Areg* is translated, *Thy1.1* is separately translated and trafficked to the surface of the cell for targeting by fluorescently conjugated antibodies (Figure 1A, see Methods). We confirmed that these mice grew normally and maintained normal histological features across multiple organ systems (Supplemental Figure 1, A and B; supplemental material available online with this article; https://doi.org/10.1172/jci.insight.187245DS1).

To analyze the fidelity of this reporter, we subjected splenic cells of *Areg^Thy1.1^* mice to a short-term PMA/ionomycin stimulation protocol (known to induce Areg production from Tregs; ref. 6) and analyzed Thy1.1 induction (live staining) on Tregs showing positive versus negative staining for AREG via the endogenous protein stain (which requires fixation/permeabilization of cells) (Figure 1B, gating strategy in Supplemental Figure 2). Unstimulated splenic cells were concurrently analyzed in this manner. Thy1.1 expression was pronounced in AREG^+^ Tregs, significantly higher than in AREG^–^ Tregs — in both stimulated and unstimulated conditions — while Thy1.1 protein expression in AREG^–^ Tregs was minimal (similar to the IgG staining control). Furthermore, Thy1.1 expression was significantly higher in stimulated AREG^+^ Tregs compared with unstimulated AREG^+^ Tregs, showing the inducibility of the Thy1.1 marker upon activation. The *Areg^Thy1.1^* reporter appeared to be less sensitive than the endogenous AREG protein stain, given that from our AREG^+^ population, only approximately 43% stained positive for the reporter. Despite this seemingly lessened staining, we note that the endogenous AREG staining method, since it is done using fixation/permeabilization, may be targeting intracellular stores that have previously been shown to form via rapid endocytosis of secreted Areg (13, 14). The endogenous stain also involves biotin-streptavidin mediated amplification of signal, which may overreport levels of Areg in these cells. Thus, we feel that the lower level of staining seen here with our reporter likely represents a more accurate accounting of Areg-producing Tregs.

To enable the isolation of live Tregs, this strain was then crossed with *Foxp3^GFP^* mice to create *Foxp3^GFP^* *Areg^Thy1.1^* mice. Importantly, upon sorting and subjecting Tregs to a long-term cytokine/TCR stimulation protocol, we saw no differences in proliferation or AREG production between Tregs from *Foxp3^GFP^* mice and *Foxp3^GFP^* *Areg*^Thy1.1^ mice, demonstrating normal functionality of Tregs harboring the *Areg^Thy1.1^* allele (Supplemental Figure 1C, gating strategy in Supplemental Figure 2).

Areg production by lung Tregs during influenza A virus (IAV) infection in mice has been previously shown by our group to be critical for proper tissue repair via signaling to a mesenchymal cell intermediate (6, 15). The bleomycin model of sterile lung injury also induces high levels of Areg-producing Tregs, as observed in previously published datasets (16, 17) and verified by our group at the protein level (data not shown). These models of lung pathology both involve extensive alveolar damage, a protracted immune response, and expansion of Tregs. However, they differ in important and complementary ways with regard to method of injury, immunostimulatory antigens, and fibrosis induction (15, 18). We thus focused our experiments on these models (Figure 1C) and validated that *Foxp3^GFP^* *Areg^Thy1.1^* mice exhibited similar disease kinetics for both the IAV and bleomycin models compared with *Foxp3^GFP^* animals, as quantified by weight loss and body temperature (IAV and bleomycin), blood oxygen saturation (IAV), and lung Pdgfra^+^ mesenchymal cell induction of α–smooth muscle actin as a fibrosis indicator (bleomycin) (Supplemental Figure 1, D and E, gating strategy in Supplemental Figure 3A).

In live lung Tregs from *Foxp3^GFP^* *Areg^Thy1.1^* mice treated with either IAV or bleomycin, we found a substantial increase in staining for Thy1.1 when compared with control saline-treated mice at 8 days post-instillation (dpi) for IAV and 14 dpi for bleomycin (Figure 1D, gating strategies for Tregs and other cell types in Supplemental Figure 3A). We additionally analyzed other cell types for Thy1.1 production during these models, finding negligible levels of induction for endothelial cells, mesenchymal cells, and myeloid cells (Supplemental Figure 3B). Among lymphoid cells besides Tregs, innate lymphoid cells — previously shown to produce Areg (19) — displayed extensive Thy1.1 expression; no other lymphoid cell types showed appreciable expression (Supplemental Figure 3B). Additionally, consistent with prior reports that epithelial cells are a major tissue source of Areg (20, 21), lung epithelial cells showed extensive expression of Thy1.1 (Supplemental Figure 3B). These characterizations of our reporter mouse verify that it provides an accurate and sensitive way to detect Areg-producing Tregs and other cell types in live sortable cells.

### Bulk RNA-Seq analysis of Areg-producing and non-Areg-producing lung Tregs from IAV- or bleomycin-treated mice.

**Figure 2:**
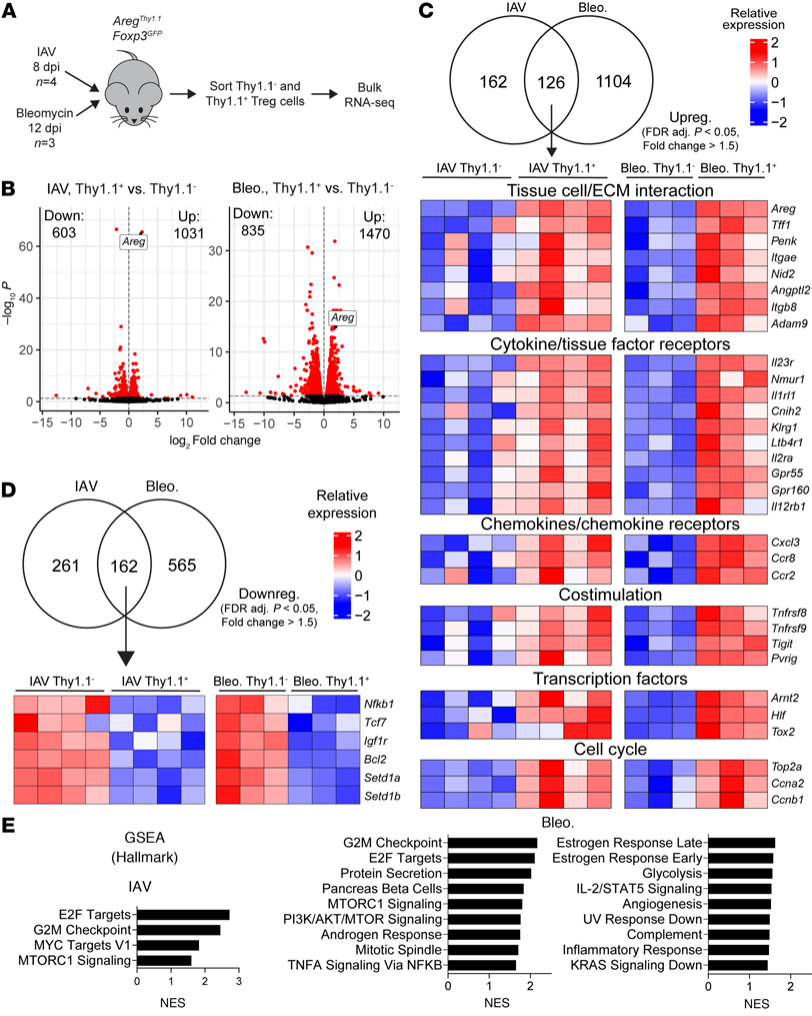
Bulk RNA-Seq of Areg-producing and non-Areg-producing lung Tregs from IAV- or bleomycin-treated mice. (**A**) Schematic of bulk RNA-Seq experiments using Thy1.1^+^ vs. Thy1.1^–^ lung Tregs from IAV and bleomycin models. (**B**) Volcano plots of differentially expressed genes (DEGs) for Thy1.1^+^ vs. Thy1.1^–^ lung Tregs from bulk RNA-Seq, from IAV and bleomycin models. Red dots on volcano plots: significant DEGs (FDR adj. *P* value < 0.05). No fold-change cutoff. Numbers of significantly upregulated and downregulated genes indicated on plots. (**C**) Top: Venn diagram showing shared genes between IAV and bleomycin model comparisons that are significantly upregulated with a fold-change induction of ≥1.5. Bottom: Heatmap of select shared genes, sorted by gene category; corresponding samples in each group are paired samples of Thy1.1^+^ vs. Thy1.1^–^ Tregs from the same mouse. (**D**) Top: Venn diagram showing shared genes between IAV and bleomycin model comparisons that are significantly downregulated with a fold-change induction of ≥1.5. Bottom: Heatmap of select shared genes; corresponding samples in each group are paired samples of Thy1.1^+^ vs. Thy1.1^–^ Tregs from the same mouse. (**E**) Pathway analysis using GSEA, in full gene signatures from IAV or bleomycin datasets depicted in **B** (from Hallmark curated gene sets). All pathways displayed are significant at FDR *q* value < 0.05. NES, normalized enrichment scores.

We utilized our reporter mouse to sort live Thy1.1^–^ (Areg-non-producing) and Thy1.1^+^ (Areg-producing) lung Tregs from the IAV and bleomycin models and performed bulk RNA-Seq (Figure 2A, gating strategy in Supplemental Figure 2). For these models, time points of 8 dpi for IAV and 12 dpi for bleomycin were chosen, as they represent the relative peaks of Treg expansion/Areg expression in lung tissue. Differential expression analysis between Thy1.1^–^ and Thy1.1^+^ Tregs undergoing the IAV model exhibited 1,634 total differentially expressed genes (DEGs) using paired analysis (Figure 2B and Supplemental Table 1). For the bleomycin model, the same comparison exhibited 2,305 total DEGs (Figure 2B and Supplemental Table 2). Validating the efficacy of our reporter, *Areg* was a top upregulated DEG in each dataset.

At a more stringent fold-change cutoff (FC > 1.5), we found that 126 upregulated DEGs were common between Thy1.1^–^ and Thy1.1^+^ Tregs in the IAV and bleomycin models (Figure 2C). This shared gene signature, by virtue of its commonality across both models and the relatively late time points used (i.e., after initial tissue damage and recruitment of Tregs), are potentially related to Treg activities associated with reparative cellular processes. Focusing on certain types of genes within this signature, we found that several genes for tissue cell/ECM interaction mediators, receptors for various cytokines and tissue factors, and chemokines/chemokine receptors were significantly altered across both models. Significantly increased presence of transcription factors *Arnt2*, *Hlf*, and *Tox2* was also seen across models, indicating an altered gene-regulatory state of Areg-producing cells. Interestingly, several genes for costimulatory molecules (*Tnfrsf8* [CD30], *Tnfrsf9* [4-1BB], *Tigit*, and *Pvrig*) were commonly differentially expressed in both models; costimulation of Tregs has not to our knowledge been previously identified as a mediator of tissue repair functionality.

We also probed commonly downregulated DEGs in these datasets (FC > 1.5) (Figure 2D). *Nfkb1*, encoding the p105/p50 subunit of the NF-κB complex, was counterintuitively downregulated (as Areg-producing Tregs are expected to undergo more activation/NF-κB signaling), possibly as a result of negative feedback following sustained activation of Thy1.1^+^ Tregs at these points (22). *Tcf1* transcription factor activity in Tregs has been shown to suppress Foxp3-activated gene programs (23), so its common downregulation in these datasets may point to a stabilization of Foxp3-induced core functional programs in Thy1.1^+^ Tregs. *Igf1r* has been shown to promote inflammatory Th17-like skewing in Tregs (24), implying Thy1.1^+^ Tregs are protected from this phenotype. *Bcl2* is a widely appreciated apoptosis inhibitor (25), so this decrease across datasets may indicate an increase in apoptosis vulnerability in Thy1.1^+^ Tregs. However, this could also relate to preferential enrichment of certain apoptosis-prone cells in Thy1.1^+^ sorted Tregs. Finally, *Setd1a* and *Setd1b* encode highly homologous histone methyltransferases that maintain epigenetic effects biasing toward specific T cell lineages (26).

Gene set enrichment analysis (GSEA) for these Treg gene signatures showed several notable pathways (Figure 2E). MTORC1 Signaling was the only signaling-associated pathway found to be enriched in both models. The MYC Targets V1 pathway was found to show significant alteration only in the IAV context (FDR *q* value < 0.05), while the PI3K/AKT/MTOR Signaling, TNFA Signaling Via NFKB, and IL-2/STAT5 Signaling pathways, among others, were significantly altered only in the bleomycin context.

Several recent studies have identified substantial heterogeneity in Tregs in tissues, including in the lung (27, 28). We reasoned that bulk sorting of lung Tregs for this RNA-Seq dataset may be capturing different proportions of subpopulations of Tregs between Thy1.1^–^ or Thy1.1^+^ cells. This possibility is underscored by the fact that several cell cycle genes (including *Top2a*, *Ccna2*, and *Ccnb1*) were upregulated in both the IAV and bleomycin Thy1.1^+^ Tregs (Figure 2C) and the fact that several of our significantly altered pathways represent cell cycle–associated processes (i.e., G2M Checkpoint, E2F Targets, and Mitotic Spindle) (Figure 2E); this may allude to the potential inclusion of more proliferating cells in sorted Thy1.1^+^ cells, which could have an outsized impact on gene signatures unrelated to their functional activity. Furthermore, significant downregulation of *Bcl2* shared between datasets could indicate the elevated presence of terminally differentiated, apoptosis-prone Treg subtypes contributing disproportionately to these gene signatures. Thus, we chose to move forward with a single-cell RNA-Seq (scRNA-Seq) approach to account for this heterogeneity.

### scRNA-Seq analysis of Areg-producing and non-Areg-producing lung Tregs from IAV- or bleomycin-treated mice.

**Figure 3:**
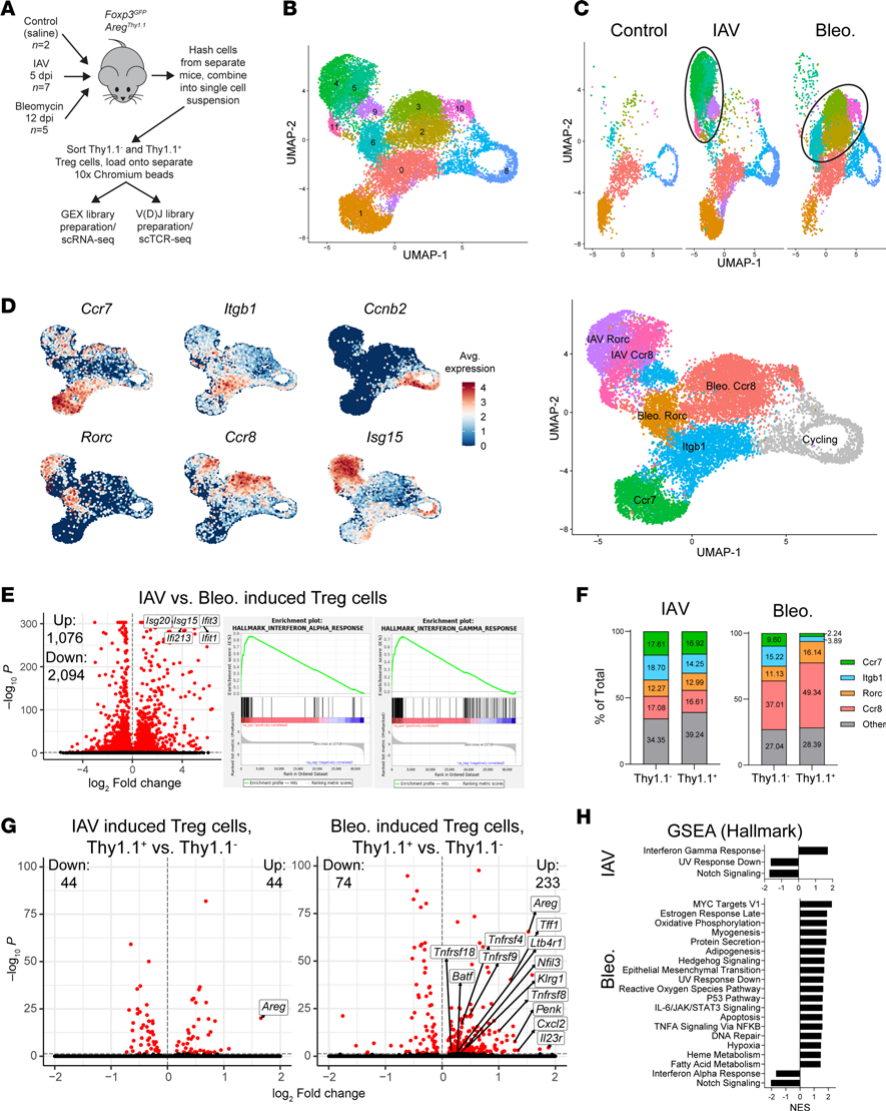
scRNA-Seq of Areg-producing and non-Areg-producing lung Tregs from IAV- or bleomycin-treated mice. (**A**) Schematic of scRNA-Seq experiments using Thy1.1^+^ vs. Thy1.1^–^ lung Tregs from IAV and bleomycin models (and control saline-treated, all Thy1.1^–^). (**B**) UMAP of clustered cells from sc gene expression analysis. (**C**) UMAP from **B**, split by treatment status of Tregs. Circles highlight groups that are largely specific to each type of tissue damage model (IAV or bleomycin). (**D**) Left: Feature plots of select genes uncovered by marker gene analysis of groups from **B**. Using these genes and/or by presence in a specific model (IAV or bleomycin), identities were assigned to groups of cells (reassigned UMAP on right). (**E**) Left: Volcano plot of DEGs between induced Treg subgroups from the bleomycin model (“Bleo. Rorc” and “Bleo. Ccr8” combined) vs. from the IAV model (“IAV Rorc” and “IAV Ccr8” combined). Red dots: significantly differentially expressed (FDR adj. *P* value < 0.05). No fold-change cutoff. Numbers of significantly upregulated and downregulated genes indicated on plots. Right: Enrichment plots of the top 2 significant pathways (FDR *q* value < 0.05) from GSEA of this gene signature, using Hallmark curated gene sets. (**F**) Proportions of each assigned subgroup in Thy1.1^+^ vs. Thy1.1^–^ Tregs in each model (Supplemental Figure 2E, separate reclustering from each model, assignment to subgroups in these plots). (**G**) Volcano plots of DEGs from Thy1.1^+^ vs. Thy1.1^–^ induced Tregs (Ccr8 and Rorc subgroups combined) from IAV and bleomycin models. Red dots: significant DEGs (FDR adj. *P* value < 0.05). No fold-change cutoff. Numbers of significantly upregulated and downregulated genes indicated on plots. (**H**) Pathway analysis using GSEA, in full gene signatures from IAV or bleomycin datasets depicted in **G** (from Hallmark curated gene sets). All pathways displayed are significant at FDR *q* value < 0.05.

To resolve heterogeneity within the Treg population, we performed scRNA-Seq on sorted Thy1.1^–^ and Thy1.1^+^ Tregs from the lungs of mice undergoing the IAV and bleomycin models, as well as Tregs from control mice (saline-treated) (all Thy1.1^–^) (Figure 3A, gating strategy in Supplemental Figure 2). We chose a different point from the bulk RNA-Seq dataset for the IAV model (5 dpi), since this has previously been shown to be the time when Tregs are the dominant source of Areg in lung tissue (6); the same 12 dpi time was used for the bleomycin model as in the bulk RNA-Seq dataset. Cells from separate mice were hashed to permit identification of cells on a per-mouse basis for analyses. Notably, Thy1.1^–^ and Thy1.1^+^ cells were run in parallel on separate lanes, for us to be able to identify these cells without a separate round of cell hashing. In doing so, we included roughly similar numbers of Thy1.1^–^ and Thy1.1^+^ cells to give us the ability to better determine heterogeneity and representation within the Thy1.1^+^ group. However, the consequence of this strategy is that cell counts from the groups in these experiments are not reflective of the actual tissue proportions of Tregs from different subtypes; thus, we did not attempt to perform analyses of numbers of Tregs of each subtype from these datasets.

Upon initial analysis using the Seurat platform (29), we first noted that most of the cells showed high expression levels of *Foxp3* and clustered in the same area of uniform manifold approximation and projection (UMAP) space, verifying their identity as Tregs, with a much smaller subcluster showing markers for other cell types (epithelial cells [*Sftpc*], endothelial cells [*Cldn5*], macrophages [*Chil3*]) (Supplemental Figure 4A); this contaminating subcluster was excluded from further analyses. To verify that our *Areg^Thy1.1^* reporter mouse was effective in allowing us to preferentially isolate Areg-producing Tregs, we queried *Areg* gene expression in total Thy1.1^–^ and Thy1.1^+^ Tregs and found that it was highly increased on a per-mouse basis, again validating the efficacy of our reporter (Supplemental Figure 4B). Upon reclustering with contaminating cells excluded, we found that Tregs were partitioned into 11 subclusters (Figure 3B). Strikingly, when separating out control, IAV-infected, and bleomycin-treated Tregs, we found that certain clusters were present across all conditions, while other clusters appeared to be largely specific to IAV infection or bleomycin induction (circled in Figure 3C).

We then assessed each population for various marker genes, assigning cell populations based on certain genes with specific expression as well as the damage model of origin (Figure 3D). The clusters marked by *Ccr7*, *Itgb1*, and *Ccnb2* were present across all conditions, including controls. *Ccr7* expression has previously been indicated as a marker for naive Tregs (30). While mouse lungs used to isolate Tregs underwent perfusion prior to lung processing and sorting, flow cytometry experiments with mouse lungs prepared in the same way, with intravenous (i.v.) labeling to mark circulating cells, indicated that some blood Tregs remained in lung tissue even after perfusion (Supplemental Figure 4C). Thus, we concluded that our “Ccr7” subset is most likely circulating naive Tregs. Since the *Itgb1*^+^ Treg population was present in control mice in addition to mice undergoing damage models, and was located closest to *Ccr7*^+^ circulating Tregs in UMAP space, we chose to interpret the “Itgb1” group as the baseline tissue-adapted lung Treg population seen in previous datasets of lung Tregs from healthy mice (27). *Ccnb2*^+^ Tregs demonstrated high expression of cell cycle genes (termed “Cycling” in our group labels) and were therefore excluded from further analysis. One of the groups that appeared only in the settings of tissue injury was marked by the *Rorc* gene, encoding transcription factor RORγt associated with Th17 cells and Tregs of the gastrointestinal (GI) tract (31). A recent report indicates that RORγt^+^ Tregs can migrate from the GI tract to the lungs under certain conditions (32), though other reports have indicated that they can be derived peripherally at locations other than the GI tract (33). The other damage-induced group was marked by the gene *Ccr8*, which has been previously described as a marker for tissue-adapted, Th2-like Tregs (34); Tregs with a similar signature have been described in lung tumors and other types of cancer (35, 36).

We looked at the expression of several known Treg- and T cell–related genes to gain a better understanding of the nature of these subgroups (Supplemental Figure 4D). *S1p1r*, the receptor that allows naive T cells to follow cues for recruitment to tissue sites, was highest in the Ccr7 subgroup, verifying their identity as naive, likely circulating Tregs. Classical Treg immunosuppression mediators *Il10*, *Tgfb1*, and *Ctla4* showed generally higher expression in tissue-resident subsets (Itgb1, Rorc, and Ccr8), implying that these groups may simultaneously perform immunosuppression and tissue repair functions. We further assessed Treg subgroup expression of master Th cell transcription factors *Tbx21* (T-bet, characteristic of type 1/Th1 immunity), *Gata3* (characteristic of type 2/Th2 immunity), and *Rorc* (RORγt, characteristic of type 3/Th17 immunity) (37). We found that *Tbx21* was not strongly expressed in any subgroup, while Ccr8 and Rorc subgroups had strongest expression of *Gata3* and *Rorc*, respectively. Finally, *Ikzf2* (encoding transcription factor Helios) has been proposed to be elevated in thymically derived Tregs in comparison with peripherally derived Tregs (38). We saw heightened *Ikzf2* expression on Itgb1 and Ccr8 subgroups, with comparably lower expression on Rorc subgroups in each model, suggesting that the Itgb1 and Ccr8 subgroups may be thymically derived, whereas Rorc subgroups may be peripherally induced.

Notably, the IAV- and bleomycin-induced Tregs (i.e., not present in control mice) (Figure 3C) each consisted of 2 main clusters marked by similar genes (*Ccr8* and *Rorc*) (Figure 3D). To ascertain why these IAV- and bleomycin-specific cells did not cluster in similar UMAP space despite this seemingly analogous gene expression pattern, we directly compared gene expression in bleomycin-induced versus IAV-induced Tregs (Ccr8 and Rorc clusters combined) (Figure 3E and Supplemental Table 3). We found that the primary axis of difference between these cells was the induction of interferon-inducible genes, which may be expected from Tregs in the interferon-enriched environment of IAV infection; this was also apparent from clear expression differences of *Isg15* between these clusters (Figure 3D). However, the similar bifurcation of IAV- and bleomycin-induced Tregs into Ccr8 and Rorc subsets indicates that in either model, newly emergent lung Tregs are likely of similar subtypes.

To gain greater clustering resolution on induced Treg subsets, we performed separate clustering on cells only from the IAV or bleomycin models (Supplemental Figure 4E). This allowed greater specificity in defining the primary Treg subgroups (Ccr7, Itgb1, Rorc, and Ccr8) seen in each model. Additionally, there were cells that showed a lack of markers for our specific subgroups, which were labeled as “Undefined/Intermediate” populations and excluded from future analyses. Interestingly, when we performed separate clustering on Tregs from control mice (Supplemental Figure 4E), beyond the Ccr7, Itgb1, and Cycling groups visible in Figure 3C, we also detected small groups of *Rorc*- and *Ccr8*-expressing cells.

To determine if the proportions of subtypes of Tregs seen in this analysis are related to their Areg production status, we analyzed the subgroup composition of Thy1.1^–^ and Thy1.1^+^ cells (Figure 3F). In the IAV model, there were only minor changes in the composition of each subset of Tregs (no changes > 5%). However, in the bleomycin group, we found that there were substantial increases in the Rorc and Ccr8 subgroups in Thy1.1^+^ Tregs compared with Thy1.1^–^, with corresponding reductions in the Ccr7 and Itgb1 subgroups.

Referring to a major advantage compared with the bulk RNA-Seq studies we conducted, the subclustering of baseline, proliferating, and damage-induced Tregs from these datasets allowed us to strictly isolate damage-induced cells for comparison between Thy1.1^–^ and Thy1.1^+^ populations (unlike in the bulk RNA-Seq dataset, where potential heterogeneity between sorted cell populations seemed to alter our DEG signatures/pathway analysis). With this in mind, we performed DEG analysis on Thy1.1^–^ and Thy1.1^+^ cells from induced Treg subsets only (Ccr8 and Rorc clusters combined), from both the IAV and bleomycin datasets (Figure 3G and Supplemental Tables 4 and 5). We found that both models exhibited altered gene signatures between these cells, with a higher level of transcriptional alteration in the bleomycin comparison versus the IAV comparison. The bleomycin gene signature includes several genes identified previously in our bulk RNA-Seq dataset, including known Treg repair mediators (*Areg*, *Tff1*, *Penk*) (10, 39), tissue factor receptors (*Klrg1*, *Il23r*, *Ltb4r1*), and costimulation receptors (*Tnfrsf8*, *Tnfrsf9*); this signature includes additional costimulation receptors that were unique to this more precise analysis (*Tnfrsf4*, *Tnfrsf18*). Additionally, chemokine *Cxcl2* is strongly induced in this context, pointing to a potential role for reparative Tregs in recruiting a specific immune milieu to the site of tissue injury. Transcription factors *Batf* and *Nfil3*, previously identified as important for tissue-adapted identity in Tregs (40, 41), were also upregulated.

Pathway analysis of these gene signatures revealed only 3 significantly altered pathways in the IAV scenario, while there were 20 significantly altered pathways in the bleomycin comparison (Figure 3H). Among the pathways revealed by this approach, several (Hedgehog Signaling, IL-6/JAK/STAT3 Signaling, Reactive Oxygen Species Pathway, and Fatty Acid Metabolism) are modes of signaling ascribed to reparative Tregs in this context that we believe to be novel. Interestingly, significant downregulation of the Notch Signaling pathway was apparent in both the IAV and bleomycin datasets; Notch signaling has recently been shown to be antagonistic toward lung Treg repair activity/Areg production (42).

As discussed in the next section, we found that both our IAV and bleomycin Tregs from this analysis showed some degree of clonal expansion but not enough for substantial analysis of expanded Treg clones. Thus, we added an additional dataset to our investigations — analysis of bleomycin-induced lung Tregs at 21 dpi (Supplemental Figure 5A), when we anticipated that Treg clonal expansion would be more pronounced. Gene expression analyses gleaned from scRNA-Seq of this new dataset yielded similar results to those shown for the bleomycin 12 dpi dataset, with similar clustering of Treg subsets (Supplemental Figure 5, B–D), further increased overrepresentation of Thy1.1^+^ cells in induced Treg subgroups (Supplemental Figure 5E), and a similar gene signature when comparing Thy1.1^–^ and Thy1.1^+^ cells in the induced Treg subgroups (Supplemental Figure 5F and Supplemental Table 6). In comparison to the bleomycin 12 dpi dataset, significant DEGs at this later time point (when Tregs have been maintained in tissue for a longer duration) included similar transcription factors to those seen in our bulk RNA-Seq (*Arnt2*, *Tox2*), further implicating these in the induction of a tissue-reparative program in Tregs.

### scTCR-Seq of Areg-producing and non-Areg-producing lung Tregs from IAV- or bleomycin-treated mice.

**Figure 4:**
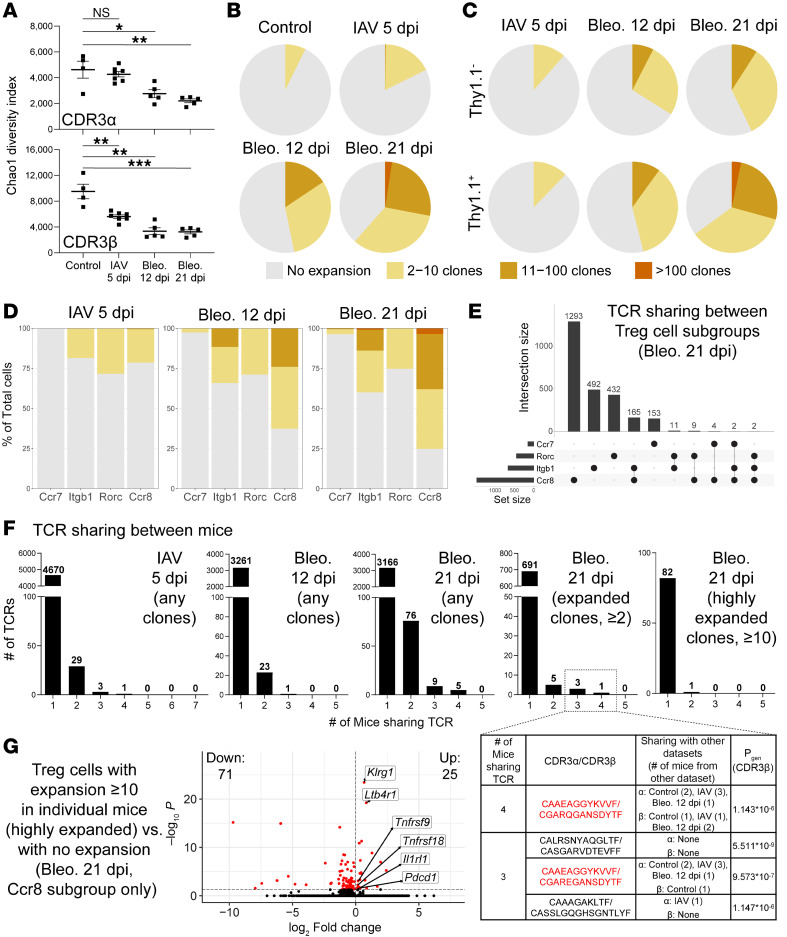
scTCR-Seq of Areg-producing and non-Areg-producing lung Tregs from IAV- or bleomycin-treated mice. (**A**) Chao1 diversity scores for lung Treg TCR repertoires (separate analysis for CDR3α and CDR3β nucleotide-level sequences) of individual mice across different datasets. (**B**) Pie charts representing the clonal expansion status of Tregs within each dataset using nucleotide-level CDR3α/CDR3β sequences. (**C**) Pie charts as in **A**, broken down by Thy1.1^–^ vs. Thy1.1^+^ status of Tregs (control mice not included, due to their being all Thy1.1^–^). (**D**) Stacked bar plots representing the clonal expansion status of Tregs within each dataset using nucleotide-level CDR3α/CDR3β sequences, subdivided by subgroup as determined from previous clustering (Supplemental Figure 4E and Supplemental Figure 5D). Cycling and undefined/intermediate subgroups not included. (**E**) UpSet plot depicting sharing of nucleotide-level CDR3α/CDR3β sequences between subgroups of Tregs from bleomycin 21 dpi. Connections between dots indicate sharing between subgroups. (**F**) Sharing of amino acid–level CDR3α/CDR3β sequences between mice in each treatment group. Numbers over bars indicate total number of TCRs shared by *n* mice. Whether graphs consider any clones (including unexpanded, single clones), expanded clones (≥2 clones in individual mice), or highly expanded clones (≥10 clones in individual mice) is indicated. Inset/table: specific CDR3α/CDR3β sequences that are expanded (≥2 clones in each mouse) in at least 3 mice from bleomycin 21 dpi, sharing with any clones from the other treatment datasets, and generation probabilities (P_gen_) (calculated using the OLGA algorithm). Red: TCR clones found in separate reports (see Results). (**G**) Volcano plot of DEGs between highly expanded Tregs (≥10 clones in any individual mouse at the nucleotide CDR3α/CDR3β level) vs. unexpanded Tregs, in the bleomycin 21 dpi Ccr8 subgroup. Red dots: significantly differentially expressed (FDR adj. *P* < 0.05). No fold-change cutoff. Numbers of significantly upregulated and downregulated genes indicated on plots. Mean ± SEM displayed on graphs. Statistical analysis was done using Bonferroni’s multiple comparisons test. *: 0.01 < *P* < 0.05, **: 0.001 < *P* < 0.01, ***: 0.0001 < *P* < 0.001.

We simultaneously evaluated the TCR repertoire of tissue Tregs during these disease models at single-cell resolution. On a per-mouse basis, when evaluating all complementarity-determining region (CDR) 3α and CDR3β nucleotide-level sequences, TCR diversity of Tregs in each dataset was highest in control mice, while a decrease was seen in IAV 5 dpi mice (significant for CDR3β only), with an even further decrease seen in bleomycin-treated mice (at both 12 dpi and 21 dpi) (significant for both CDR3α and CDR3β) (Figure 4A). Next, we evaluated Treg clonal expansion between each separate model/time point, at the level of CDR3α/CDR3β paired nucleotide TCR sequences (Figure 4B). As expected, control mice showed minimal clonal expansion with no clones expanded to more than 10 cells. Tregs from IAV-treated mice (5 dpi) also showed minimal expansion with only a small amount of clones expanded more than 10, likely as a consequence of the early nature of this time. Within the bleomycin datasets, we saw several clones expanded to more than 10 at 12 dpi, while this was even further pronounced at 21 dpi (with several clones expanded to >100). We additionally evaluated TCR expansion in Thy1.1^–^ and Thy1.1^+^ cells from each separate model/time point (Figure 4C). While there were slight increases in expansion of clones in Thy1.1^+^ Tregs compared with Thy1.1^–^ in the IAV 5 dpi and bleomycin 12 dpi datasets, this difference was profoundly increased in the bleomycin 21 dpi dataset, where the majority of Thy1.1^+^ cells were expanded. These data indicate that Tregs undergo progressive clonal expansion in a sterile model of lung damage (bleomycin) and that Areg-producing reparative Tregs show features of heightened clonal expansion.

Simultaneous evaluation of scRNA-Seq and scTCR-Seq additionally allowed us to examine TCR expansion in specific Treg subsets. These results indicate that the Ccr8 subset in the bleomycin model (at either time point) contained the greatest number of expanded Tregs, while there did not appear to be major differences among Itgb1, Rorc, and Ccr8 subsets in the IAV dataset (Figure 4D). With respect to clonal sharing between subgroups, we found that the highly expanded Ccr8 TCRs in the bleomycin 21 dpi dataset primarily were shared with the Itgb1 subgroup, with hardly any sharing between other subgroups (Figure 4E). This may indicate a developmental trajectory between these subgroups, wherein Tregs first entering tissue are of the Itgb1 phenotype, later converting to a Ccr8 phenotype after further clonal expansion; this interpretation is supported by the similar expression of *Gata3* and *Ikzf2* between the Itgb1 and Ccr8 subgroups (compared with Rorc) (Supplemental Figure 4D).

Next, we sought to assess TCR sharing between different mice undergoing each damage model/time point (here using CDR3α/CDR3β paired amino acid TCR sequences to account for identical TCRs expanded from slightly different nucleotide sequences between mice) (Figure 4F and Supplemental Table 7). For the IAV and the bleomycin 12 dpi dataset, no TCRs were shared among all mice analyzed, though there was a low degree of sharing between 2–4 mice in each model. However, for the bleomycin 21 dpi dataset, 14 TCRs were shared between at least 3 mice. We then heightened the stringency of this analysis to ascertain the prevalence of expanded TCRs (≥2 clones/mouse) across mice in the bleomycin 21 dpi dataset. When looking at only expanded TCRs, we found that 4 were shared by at least 3 mice from this dataset (highlighted in inset/table in Figure 4F). To query whether this TCR-sharing pattern is apparent among TCRs with the highest degree of clonal expansion, we then performed this same analysis on highly expanded clones from the bleomycin 21 dpi dataset (≥10 clones/mouse) and found that none were shared by all or even 3–4 mice; only 1 was shared by 2 mice.

This latter analysis seems to imply stochastic clonal expansion of Tregs, with different highly expanded clones dominating in different mice in a manner indicative of localized response to microenvironment signals (e.g., alarmins). On the other hand, TCR diversity generation in vivo has incredible breadth, and we have analyzed a limited amount of Tregs from each mouse in this study because of the limitations of the methods used here (i.e., an average of 1,184 complete TCR sequences per bleomycin-treated mouse lung from a total estimated Treg number of 50,000–100,000 per bleomycin-treated mouse lung). Thus, the finding that several lower level expansion TCRs are shared (inset/table in Figure 4F) may instead be evidence that a limited subset of clones can expand in response to tissue damage.

To this effect, for 2 of the expanded/shared clones with highly similar sequences (red in inset/table in Figure 4F), a separate study identified the same CDR3α and CDR3β sequences in highly expanded Treg clones from a lung cancer model (43) (non-peer-reviewed doctoral thesis), and another identified them as shared clones between a different lung cancer model and an allergic asthma model (44) (non-peer-reviewed doctoral thesis). Additionally, the CDR3β of one of these clones was identified as highly expanded in a model of silica-induced lung fibrosis (45). Since these clones appear in alternative models of lung tissue damage by independent groups, one could infer that these clones are most likely to be damage induced and potentially responding to a tissue antigen. However, in one of these studies (43), the authors used extensive yeast-displayed peptide-MHC libraries in an attempt to elucidate peptide antigens for these TCRs, finding that these clones seemingly show largely non-antigen-specific binding characteristics, even after sequential rounds of affinity-based selection. Furthermore, the CDR3α and β chains from these clones are highly shared among our other datasets from IAV 5 dpi, bleomycin 12 dpi, and even control mouse lung Tregs (inset/table in Figure 4F). While they do not appear as “public” CD4^+^ T cell TCRs widely expressed in C57BL/6 mouse splenic peripheral repertoires (46, 47), their presence in our other datasets and their appearance in other studies indicate that they may be public TCRs restricted to the lung environment. Consistent with this concept, these TCRs had relatively high P_gen_ (inset/table in Figure 4F) (48), indicating TCR sequences that are more likely to be generated based on V(D)J recombination patterns in a manner unrelated to antigen specificity.

Last, we used the high prevalence of expanded clones in our bleomycin 21 dpi dataset to evaluate potential gene signature differences between highly expanded Treg clones (≥10 clones in any individual mouse at the CDR3α/CDR3β paired nucleotide TCR sequence level) and unexpanded Tregs in this damage context (within the Ccr8 subset only) (Figure 4G and Supplemental Table 8). This comparison yielded a DEG signature showing upregulation in receptors for tissue factors (*Klrg1*, *Il1rl1*, *Ltb4r1*), T cell activation markers (*Pdcd1*), and costimulation receptors (*Tnfrsf9*, *Tnfrsf18*).

### Analysis of immunosuppression and tissue repair gene modules in damage-induced lung Tregs.

**Figure 5:**
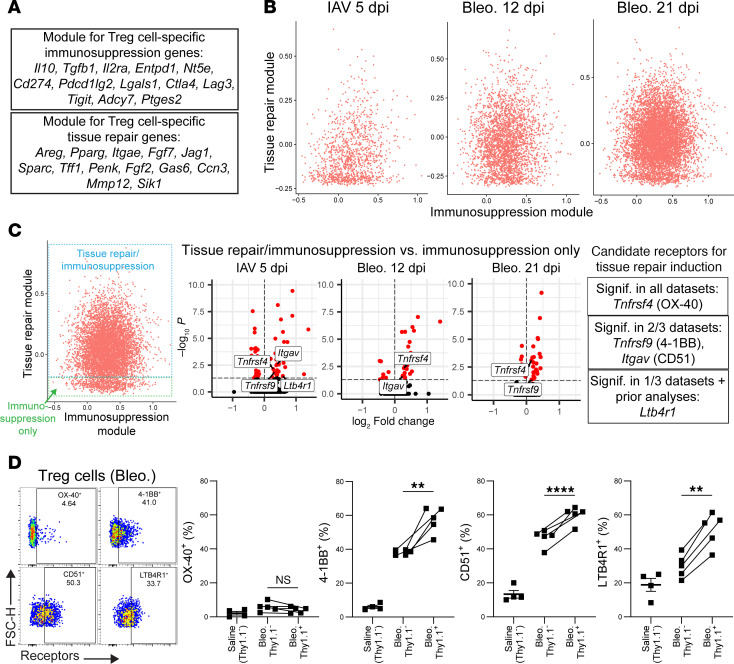
Analysis of Immunosuppression and Tissue repair gene modules of lung Tregs from IAV- or bleomycin-treated mice. (**A**) Immunosuppression and Tissue repair modules of genes (Treg-specific), established from literature. (**B**) Immunosuppression and Tissue repair module scores were calculated for cells in the Ccr8 subgroup of each treatment dataset, then plotted against each other. (**C**) Left: Subclusters with differing patterns of module expression are indicated by boxes on plot. Middle: Volcano plots of DEGs between groups indicated at left for each treatment dataset. Red dots: significantly differentially expressed (FDR adj. *P* value < 0.05). No fold-change cutoff. Genes encoding receptors of interest for activation of tissue repair function are indicated on graphs and in the summary on the right. (**D**) Protein expression assessed by flow cytometry for lung Tregs (saline or bleomycin 14–15 dpi) for each of the candidate receptors in saline (*n* = 4) and bleomycin mice (*n* = 5, separated into Thy1.1^–^ and Thy1.1^+^ cells). Representative plots shown on left for combined Thy1.1^–^/Thy1.1^+^ Tregs (bleomycin 15 dpi). Gating based on individual FMO controls. LTBR1^+^ Tregs were gated from only CD45 i.v. negative cells for technical reasons from mouse-on-mouse staining (see Supplemental Methods). All values included from 2 experiments. Statistical analysis for comparisons between 2 groups was done using 2-tailed paired Student’s *t* tests, between Thy1.1^–^ and Thy1.1^+^ cells from the same mice. **: 0.001 < *P* < 0.01, ****: *P* < 0.0001.

A major question since the discovery of the reparative function of Tregs has been whether distinct subsets of Tregs are responsible for immunosuppression versus tissue repair functionality. We attempted to evaluate this paradigm by querying our scRNA-Seq datasets using gene module analysis. We generated an “Immunosuppression” gene module that includes genes known to relate specifically to immunosuppressive functions in Tregs (Figure 5A) (1, 49). We also created a “Tissue repair” gene module that includes genes that have been shown in published literature to encode mediators produced by Tregs that have a direct impact on tissue repair (2), as well as the transcription factor *Pparg*, which has been demonstrated to be a marker of tissue-adapted Tregs (50) (Figure 5A). We found that our gene module for Tissue repair was primarily enriched in the Ccr8 subset in our datasets (Supplemental Figure 6A). Thus, we chose to further focus on only the Ccr8 subset for these analyses.

Cellular scores from the Immunosuppression module and the Tissue repair module were plotted against each other within the Ccr8 subset for each dataset (Figure 5B). Strikingly, we saw that 2 separate groups of cells appeared: a group that expresses immunosuppression genes but no tissue repair genes (which we called the “Immunosuppression only” group) and a group that expresses both immunosuppression genes and tissue repair genes (which we called the “Tissue repair/immunosuppression” group) (Figure 5C). Thy1.1^+^ Tregs contained higher ratios of Tissue repair/immunosuppression cells to Immunosuppression only cells on a per-mouse basis, compared with Thy1.1^–^ cells (Supplemental Figure 6B). To gather further insight into the differences between these subgroups, we performed differential expression analysis comparing them in each dataset (Figure 5C and Supplemental Tables 9–11). When analyzing these gene signatures, our focus was on discovering potential receptors on Tissue repair/immunosuppression Tregs that can potentially activate their tissue repair functionality. To this effect, we found that *Tnfrsf4*, encoding T cell costimulatory molecule OX-40, was significantly upregulated in all 3 datasets; *Tnfrsf9* and *Itgav*, encoding T cell costimulatory molecule 4-1BB and CD51 (integrin αV, a receptor for ECM molecule vitronectin), were significantly upregulated in 2/3 datasets; and *Ltb4r1*, encoding leukotriene B4 receptor (LTB4R1), was significantly upregulated in 1/3 datasets (but was additionally differentially expressed in other datasets throughout this study). These receptors — reflective of costimulation by other cells in the lung milieu (OX-40 and 4-1BB), ECM-induced tissue adaptation (CD51), or leukotriene signaling (LTB4R1) — have not been previously explored for their role in inducing tissue repair by Tregs. We verified expression of these proteins in bleomycin-induced lung Tregs (Figure 5D) and found low levels of OX-40, with higher levels of 4-1BB, CD51, and LTB4R1; furthermore, the latter 3 receptors were all significantly upregulated on Thy1.1^+^ Tregs compared with Thy1.1^–^ (Figure 5D).

### Ex vivo assessment of functional repair activity induction in damage-induced lung Tregs.

**Figure 6:**
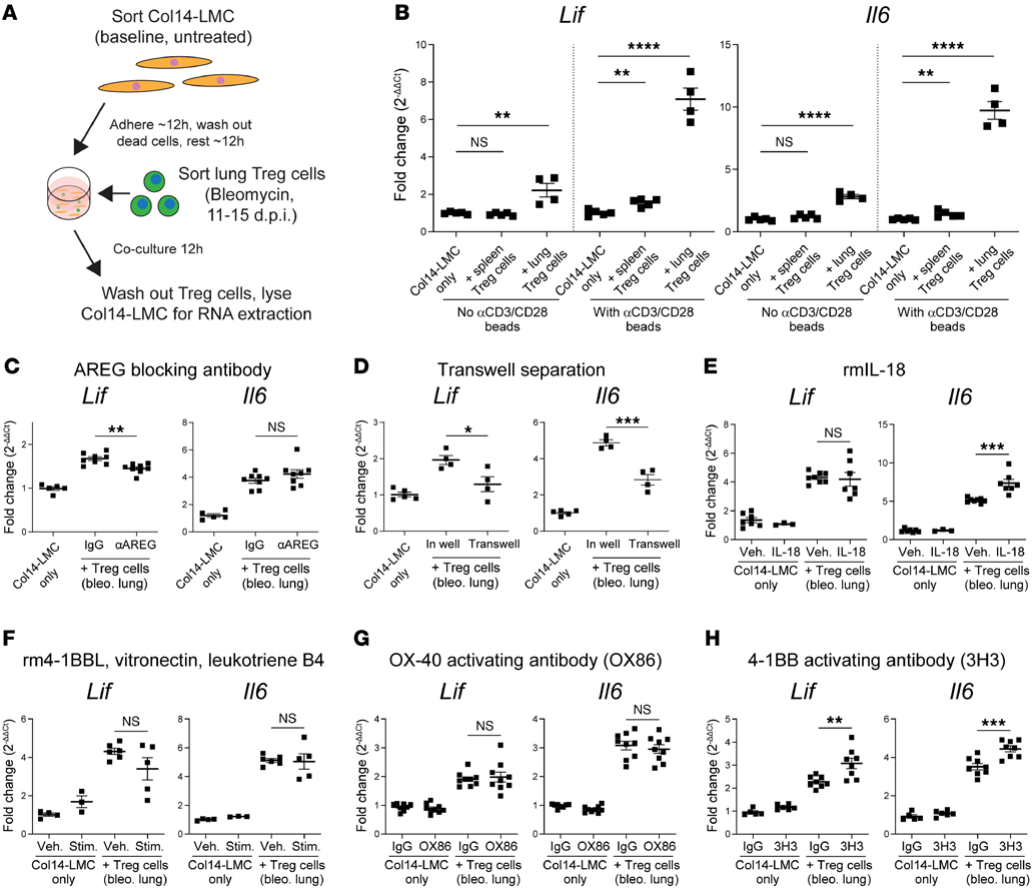
Testing of candidate receptors in functional coculture assay identifies 4-1BB agonism as tissue repair inductive. (**A**) Experimental schematic of Col14-LMC and Treg coculture. (**B**) Quantitative PCR (qPCR) for Treg-induced genes *Lif* and *Il6* in Col14-LMC following lung or spleen Treg coculture, with or without anti-CD3/CD28 (αCD3/CD28) beads for T cell activation as indicated. *n* = 4–5 per group; all values included from 2 experiments. (**C**) qPCR for *Lif* and *Il6* in Col14-LMCs following Treg coculture, with control IgG or αAREG antibody. *n* = 5–8 per group; all values included from 3 experiments. (**D**) qPCR for *Lif* and *Il6* in Col14-LMCs following Treg direct coculture or separation with a 0.4 μm Transwell insert. *n* = 4–5 per group; all values included from 3 experiments. (**E**) qPCR for *Lif* and *Il6* in Col14-LMCs following Treg coculture, with vehicle or recombinant murine IL-18 (rmIL-18). *n* = 3–8 per group; all values included from 3 experiments. (**F**) qPCR for *Lif* and *Il6* in Col14-LMCs following Treg coculture, with vehicle or a combination of rm4-1BB ligand, vitronectin, and leukotriene B4. *n* = 3–6 per group; all values included from 2 experiments. (**G**) qPCR for *Lif* and *Il6* in Col14-LMCs following Treg coculture, with control IgG or αOX-40 activating antibody (clone OX-86). *n* = 8–9 per group; all values included from 3 experiments. (**H**) qPCR for *Lif* and *Il6* in Col14-LMCs following Treg coculture, with control IgG or α4-1BB activating antibody (clone 3H3). *n* = 5–8 per group, all values included from 2 experiments. Mean ± SEM displayed on graphs. Statistical analysis was done using 2-tailed unpaired Student’s *t* tests for comparisons between 2 groups or Bonferroni’s multiple-comparison test where multiple groups were compared. *: 0.01 < *P* < 0.05, **: 0.001 < *P* < 0.01, ***: 0.0001 < *P* < 0.001, ****: *P* < 0.0001.

To address the potential for activation of tissue repair function of these receptors, we utilized an ex vivo coculture assay previously developed in our lab by which to test the tissue repair functionality of Tregs. Here, Tregs are cocultured with a subpopulation of lung mesenchymal cells (LMCs) involved in alveolar regeneration (characterized by high expression of the *Col14a1* gene encoding collagen XIV; hereafter referred to as “Col14-LMCs”) (Figure 6A, gating strategy in Supplemental Figure 2) (15). Coculture of bleomycin-induced lung Tregs promoted greater expression of *Il6* and *Lif* (another IL-6 family cytokine) from Col4-LMCs, compared with Col14-LMCs alone or splenic Tregs from bleomycin-treated mice (Figure 6B). This was further enhanced by the addition of αCD3/CD28 beads to cultures, in order to stimulate Treg activity (Figure 6B). Blocking of Areg with an αAreg antibody significantly reduced the transcription of *Lif* (but not *Il6*) in Col14-LMCs compared with IgG controls (Figure 6C). The partial nature of this inhibition indicates that there are additional non-Areg mechanisms of Col14-LMC stimulation at play. We additionally tested contact dependency of Treg-mediated Col14-LMC activity by separating Tregs from Col14-LMCs in a Transwell plate. We found that there was a partial but significant decrease in *Il6* and *Lif* transcription in Transwell-separated Col14-LMCs compared with controls (Figure 6D). The partial nature of this decrease indicates that there are likely both contact-dependent and soluble factors from Tregs that contribute to Col14-LMC stimulation.

To assess whether factors known to induce the tissue repair activity of Tregs are able to stimulate Treg-mediated Col14-LMC activity, we tested the addition of IL-18 in this assay. IL-33 was not able to be tested because it unexpectedly caused direct stimulation of Col14-LMCs (data not shown). IL-18, when added along with bleomycin-induced lung Tregs, was able to significantly increase transcription of *Il6* (but not *Lif*) in Col14-LMCs compared with vehicle controls (Figure 6E). Interestingly, this assay does not seem to be simply demonstrating nonspecific effects of Treg activation (i.e., wherein other Treg functions are also amplified), since IL-18 has previously been demonstrated to dampen Treg-mediated suppression (51).

We then turned to testing the potential repair activity–inducing ligands for the receptors uncovered from our scRNA-Seq analyses (Figure 5C). We first attempted to treat Tregs in the coculture assay with ligands for 4-1BB (recombinant mouse 4-1BBL), CD51 (vitronectin), and LTB4R1 (leukotriene B4), based on their substantial expression levels on bleomycin-induced lung Tregs (Figure 5D). The combination of these ligands was unable to confer greater Treg-induced Col14-LMC activity, based on unchanged expression of *Il6* and *Lif* (Figure 6F). While OX-40 was expressed at a comparatively lower level than the other receptors at the protein level, it showed the most consistent upregulation in our scRNA-Seq analysis of tissue repair/immunosuppression-oriented versus immunosuppression only–oriented Tregs, so we decided to test activity through this costimulator molecule as well, using an activating antibody toward OX-40. However, this approach also yielded no changes at the level of Col14-LMC transcription compared with IgG controls (Figure 6G).

Maximal 4-1BB downstream activation requires multimerization via interaction with multiple 4-1BBL proteins on the surface of costimulating cells (52), an effect that may not be occurring when recombinant 4-1BBL is used in this system as in Figure 6F. Thus, we used an activating antibody for 4-1BB that has previously shown efficacy toward T cell activation in vivo (53), to induce some level of multimerization. Using this approach, we found that this antibody was able to induce greater transcription of both *Il6* and *Lif* in Col14-LMCs when added to Treg/Col14-LMC cocultures, compared with IgG controls (Figure 6H). Similar to IL-18, activation via 4-1BB (using this same agonistic antibody) has previously been shown to decrease immunosuppressive activity by Tregs (54, 55). Thus, 4-1BB stimulation seems to specifically induce repair activity in Tregs, rather than nonspecifically inducing all functional activities.

### 4-1BB stimulation in damage-induced lung Tregs induces repair gene expression while reducing immunosuppression gene expression and reveals potentially novel mediators of Treg repair.

**Figure 7:**
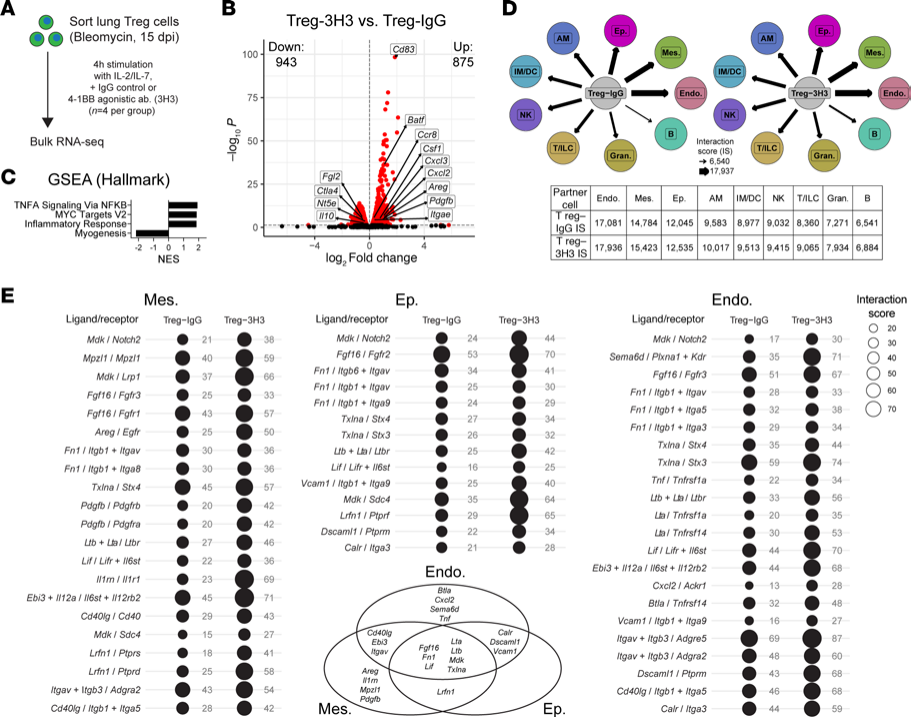
4-1BB agonism effects reparative activity induction and immunosuppression reduction in lung Tregs and identifies potential ligand-receptor interactions with structural tissue cells. (**A**) Experimental schematic for bulk RNA-Seq of bleomycin lung Tregs stimulated with 4-1BB agonistic antibody. (**B**) Volcano plots of DEGs for 4-1BB agonistic antibody–stimulated (3H3) vs. control IgG–treated lung Tregs. Red dots on volcano plots: significant DEGs (FDR adj. *P* value < 0.05). No fold-change cutoff. Numbers of significantly upregulated and downregulated genes indicated on plot. (**C**) Pathway analysis using GSEA, in full gene signature depicted in **B** (from Hallmark curated gene sets). All values displayed are significant at FDR *q* value < 0.05. (**D**) ICELLNET ligand/receptor interaction analysis of IgG-treated or 4-1BB agonistic antibody–treated lung Tregs, with lung cell types from the Mouse Cell Atlas scRNA-Seq dataset (National Center for Biotechnology Information Gene Expression Omnibus GSE108097). Arrows indicate composite interaction scores (IS) over all ligand/receptor pairs for a given pairing. Table inset: IS values for each pairing. Endo., endothelium; Mes., mesenchyme; Ep., epithelium; AM, alveolar macrophages; IM/DC, interstitial macrophages/dendritic cells; NK, NK cells; T/ILC, T cells/innate lymphoid cells; Gran., granulocytes; B, B cells. (**E**) ICELLNET bubble plots identifying all interactions between significantly upregulated genes from **B** with expressed receptors from mesenchymal cells, epithelial cells, and endothelial cells. IS for a given ligand/receptor pairing are given to the right of each bubble. Inset: Venn diagram comparing ligands found for each structural cell type.

To further explore the effect of 4-1BB agonism on reparative phenotype induction in lung tissue Tregs, we sorted Tregs from bleomycin-induced lungs, treated matched populations from separate mouse pools with IgG control or agonistic 4-1BB antibody (3H3), and performed bulk RNA-Seq (Figure 7A). Complementary to our results in the coculture assay and prior work showing decreased immunosuppression activity, we observed an increase in tissue repair gene expression (*Areg*, *Pdgfb*, *Itgae*) concomitant with a decrease in immunosuppressive gene expression (*Il10*, *Ctla4*, *Nt5e* [CD73], *Fgl2*) (Figure 7B and Supplemental Table 12); *Itgae* (CD103) on Tregs has been shown to directly interact with the lung epithelium (56), while growth factor *Pdgfb* has been found to be secreted by Tregs within the bleomycin model (57). *Ccr8*, which marked one of the induced Treg groups in our scRNA-Seq datasets, is also upregulated. *Cd83*, the top upregulated gene, has been identified as an important factor for proper differentiation and activation of Tregs (58). *Batf*, another highly upregulated gene, has been shown to serve as a critical transcriptional regulator of tissue Treg programs (40, 59). As highlighted in a recent report and several other investigations (2, 60), an alternative way in which Tregs mediate tissue repair is by signaling to macrophages; in support of this, macrophage-targeted factors *Csf1*, *Cxcl2*, and *Cxcl3* are upregulated in Tregs by 4-1BB agonism. Pathway analysis showed similar upregulated motifs to our previous analyses of reparative Tregs, with the exception of Myogenesis, which is here downregulated as opposed to upregulated in a prior dataset (Figure 7C, compare with Figure 2E and Figure 3H).

We then sought to investigate the potential of 4-1BB–mediated activation for altering the interaction ability of Tregs with other populations in the lung. To do this, we utilized the ligand/receptor interaction software framework ICELLNET, which unlike other platforms of this nature is designed for the use of bulk RNA-Seq data (61, 62). For this analysis, we incorporated lung scRNA-Seq data from the Mouse Cell Atlas to isolate pseudobulk transcriptional signatures of different mouse lung cell populations (Supplemental Figure 7A) (63). We found that 4-1BB agonism increased the interaction potential of Tregs with all cell types in the lung compared with IgG stimulation (as indicated by composite ligand/receptor interaction score; see Methods). Notably, the highest overall interaction scores were with structural cell types (mesenchymal, epithelial, endothelial) compared with immune cell types (Figure 7D).

To zoom in on potentially novel Treg–structural cell ligand/receptor pairings, we assessed pairings of significantly upregulated genes for ligands from 4-1BB agonism in Tregs with receptors on specific structural cell populations (Figure 7E). Verifying the efficacy of this approach, AREG/EGFR appeared as an enriched interaction specifically with mesenchymal cells, which is consistent with our previous study (15). These putative ligands vary in their specificity for receptors on the different structural cell types, with some identified as interacting with all three (inset in Figure 7E). Several of these ligand genes were highly expressed by certain Treg subsets from our scRNA-Seq datasets (Supplemental Figure 7B). The majority of these ligand/receptor pairings have not to our knowledge been previously investigated in the context of Treg-mediated tissue repair.

## Discussion

Transcriptomic and functional analysis of tissue repair–oriented, Areg-producing immune cells has previously been unavailable because of the inability to sort live Areg-producing cells. Our *Areg^Thy1.1^* reporter mouse addresses this deficiency, which when paired with single-cell RNA and TCR sequencing allowed us to interrogate reparative Tregs in the lung in different models of damage at multiple time points of disease progression.

We found that our bulk RNA-Seq efforts to elucidate differences in reparative Tregs in models of lung damage were hampered by heterogeneity between sorted Thy1.1^–^ versus Thy1.1^+^ cells, possibly related to the inclusion of variable amounts of different cell subtypes. Our use of scRNA-Seq to address this heterogeneity revealed several tissue subsets of Tregs in the setting of lung damage. Certain subgroups, when compared with the Tregs present in the lung in control mice, were only substantially detected during lung damage — the Ccr8 and Rorc subgroups identified here. Limiting our comparison to only these “induced” subsets of Tregs allowed us to ascertain a defined gene signature associated with reparative, Areg-producing Tregs. Several of the pathways identified in reparative Tregs have not been previously studied in relation to tissue repair. For instance, Hedgehog signaling has been shown to mediate the conversion of Tregs to a Th17-like phenotype in the context of breast cancer (64). Whether such alterations are present and meaningful in the context of tissue damage remains to be seen; this study provides the groundwork for several such lines of investigation.

There is currently a surfeit of knowledge in the field of immunology regarding the specific antigens or other tissue cues that evoke expansion of T cells in the context of sterile damage. Our work here shows that Tregs undergo progressive clonal expansion in the context of sterile lung injury (bleomycin). This may relate to increased access of reparative Tregs to certain tissue self-antigens that are exposed upon lung damage. Alternatively, the lung microbiota, while known to be less established than microbiota in other organs, such as the GI tract, is known to have an impact on the bleomycin model (65), and Treg expansion in this context could be in response to the microbiome. The fact that groups in separate facilities, likely to have dissimilar lung microbiomes, uncovered similar expanded clones to ones found here in different models of lung damage (43–45) would seem to indicate that a damage-induced self-antigen is a more likely candidate, but further studies (potentially with germ-free mice) must be done in this regard to fully address this.

The potential antigen specificity of the clonally expanded Tregs found in this study was not investigated here, but recent work has offered insight in this regard. One study (43) investigated 2 TCR clones analogous to shared/expanded clones from our bleomycin 21 dpi dataset, specifically found as expanded in Tregs in a lung adenocarcinoma model. However, as discussed in Results, their yeast-displayed peptide/MHC-II library screening was unable to uncover specific epitopes. In addition, they transduced T cells with one of these TCRs and adoptively transferred them to *Rag2*^–/–^ mice, but they failed to see greater localization to the lung, either at baseline or in a model of lung cancer. Thus, a different expanded/shared TCR from bleomycin 21 dpi may be a better candidate for a damage-induced TCR clone, based instead on lesser sharing with other datasets and lower generation probability. A fully transgenic model encoding one of the shared/expanded TCRs from our dataset may be helpful in elucidating the effect of these common lung-expanded clones across models (7, 8).

Treg costimulation by the Tnf receptor superfamily (Tnfrsf genes) is known to have several important functions in activation and proliferation (66), but its effects on initiation of reparative activity in Tregs are unknown. Our data show that in an in vitro assay for tissue repair activity, activation through 4-1BB heightens Treg signaling to lung mesenchymal target cells. Furthermore, this does not seem to be related to general activation of Tregs when stimulated through this pathway, given that other work has shown that Tregs are in fact less immunosuppressive when stimulated through this pathway (54, 55). Bulk RNA-Seq of induced lung Tregs stimulated with agonistic 4-1BB antibody verified these patterns at the gene expression level; ligand/receptor interaction analysis of this dataset highlighted the high interaction potential of lung Tregs with structural cell types in the lung and uncovered potentially novel reparative molecules produced by lung Tregs that merit further investigation. Given that other T cells can express 4-1BB (55), models wherein reparative effects of 4-1BB stimulation can be isolated specifically to Tregs in vivo would be important for further establishing a role of signaling through this pathway in tissue repair.

In conclusion, the findings from this study provide a foundation for further investigation of Treg phenotypes and clonal expansion states associated with tissue-reparative activity. Furthermore, the *Areg^Thy1.1^* knockin mouse generated for this report could be used by other groups to investigate other aspects of reparative Treg biology or other cell types that produce Areg. There are many potential therapeutic benefits to harnessing optimal tissue repair abilities by Tregs in the lung and at other tissue sites (67); we hope that this work has offered some insight into the transcriptional and cellular pathways used by reparative Tregs, which may ultimately assist in the development of these types of interventions.

## Methods

### Sex as a biological variable.

For the transcriptomic studies and lung disease induction comparisons in this report, only male mice were used; this was done based on prior work indicating that Areg-mediated repair during influenza-induced lung damage in mice shows differences between sexes (68) and that bleomycin-induced lung damage is more pronounced in males (69). All other experiments in this report utilize both male and female mice.

### Additional methods.

For detailed methods regarding “Mice”; “Bulk RNA-Seq”; “Single cell RNA- and TCR-Seq”; “Mouse lung damage models and assessment”; “Lung, spleen, and lymph node processing”; “Splenic/lymph node Treg stimulation protocols”; “CD4 T cell bead enrichment and Treg sorting”; “Flow cytometry”; “Col14-LMC negative enrichment and sorting”; “Col14-LMC/Treg co-culture”; and “RNA extraction and qPCR,” see Supplemental Methods. References uniquely in Supplemental Methods include refs. 70–78.

### Statistics.

R (version 4.2.2), Python (version 3.1), and GraphPad Prism (version 10.1.2) were used for all statistical analyses and graphing. For flow cytometry, qPCR, or TCR diversity (Chao1) analysis, 2-tailed unpaired Student’s *t* tests were used where 2 groups were compared, Bonferroni’s multiple comparisons test was used where multiple groups were compared, and 2-tailed paired Student’s *t* tests were used where data were paired from the same mouse. For longitudinal weight loss, body temperature, or blood oxygen saturation analysis, 2-way repeated measure ANOVA was used. Statistical significance was determined at *P* < 0.05, with further levels of significance reported in figure legends. Sample size estimation was determined based on previous studies. FlowJo (version 10.7.1), Microsoft Excel (version 16.0), SnapGene Viewer (version 6.0.4), Adobe Illustrator (version 24.1.2), MacVector (version 17.0), and R were used to set up experiments, analyze data, and prepare data.

### Study approval.

Animal experiments were approved by Columbia University’s Institutional Animal Care and Use Committee (protocol AC-AABT2656).

### Data availability.

Sequencing data associated with this manuscript have been deposited in NCBI’s Gene Expression Omnibus (accession numbers: GSE277226, GSE277256, and GSE292440). We report no original source code for this manuscript. Raw data from graphs included in this publication are given in a Supporting Data Values file. All other data have been included in the manuscript or supplement.

## Author contributions

LFL, KAK, AH, and NA designed research; LFL, KAK, AK, SR, and KDLSA performed research; LFL, KAK, AK, and SR analyzed data; and LFL, KAK, and NA wrote the paper. Order of co–first authorship was determined as such because of the final assembly of the manuscript by LFL.

## Supplementary Material

Supplemental data

Supplemental tables 1-12

Supporting data values

## Version 1 07/08/2025

Electronic publication
