# Supplementary material for: An amphiregulin reporter mouse enables transcriptional and clonal expansion analysis of reparative lung Tregs
Source: JCI Insight. 2025 Jul 8;10(13):e187245. doi: 10.1172/jci.insight.187245 (PMC12288904; doi:10.1172/jci.insight.187245)
Supplement: Supplemental data [file jciinsight-10-187245-s072.pdf]

Supplemental material for:

An amphiregulin reporter mouse enables transcriptional and clonal expansion analysis of reparative lung Treg cells

Lucas F. Loffredo, Katherine A. Kaiser, Adam Kornberg, Samhita Rao, Kenia de los Santos-Alexis, Arnold Han, Nicholas Arpaia

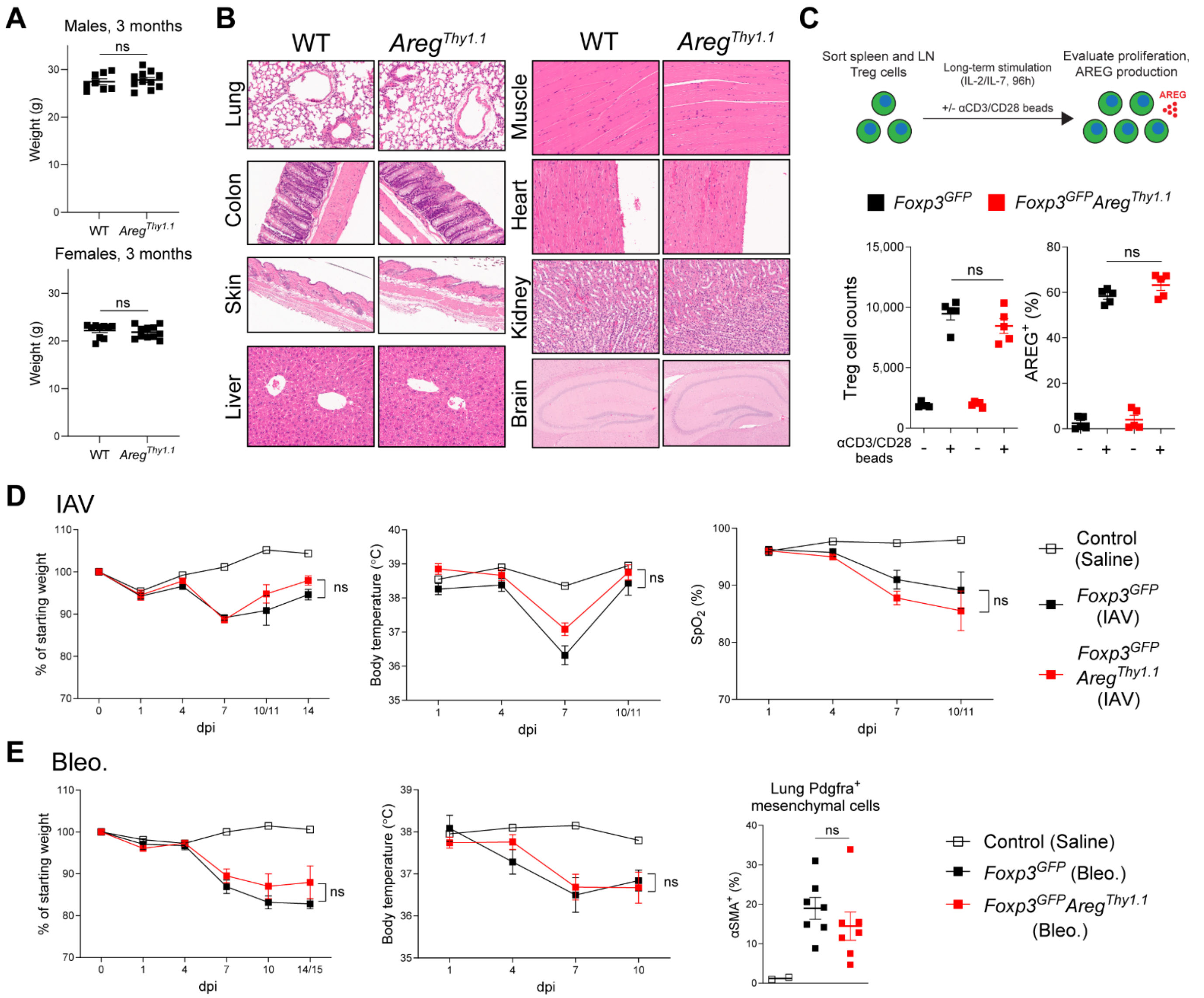

Supplemental Figure 1. Supplemental data from Figure 1.

A) Weights of WT or *Areg*$^{Thy1.1}$ mice (male or female) at 3 months of age. From 2 separate cohorts of mice of each sex. **B)** Histology from indicated organs of WT or *Areg*$^{Thy1.1}$ mice (hematoxylin & eosin staining). All sections are at 20x magnification besides the brain (5x). Representative histology shown from 2 separate experiments. **C)** Treg cells were sorted from spleen/lymph node cell suspensions from *Foxp3*^{GFP} or *Foxp3*^{GFP}*Areg*$^{Thy1.1}$ mice, then cultured for 96h with IL-2 and IL-7, with or without the inclusion of αCD3/CD28 beads (see schematic). Following this, Treg cells were counted and stained for endogenous AREG protein production. Gating strategy for Treg cell sorting in Supplemental Fig. 2. Graph contains all values from 2 separate experiments. **D)** *Foxp3*^{GFP} or *Foxp3*^{GFP}*Areg*$^{Thy1.1}$ mice were treated with IAV (PR8-H1N1, 100 TCID50, intranasal), with weight, body temperature, and blood oxygen saturation measured every 3-4 days to assess course of disease. n=5-6 mice per IAV groups, 2 mice in control group. Graphs contain all values from 2 separate experiments. dpi: days post-instillation. **E)** *Foxp3*^{GFP} or *Foxp3*^{GFP}*Areg*$^{Thy1.1}$ mice were treated with bleomycin (1 mg/kg, intratracheal), with weight and body temperature measured every 3-4 days to assess course of disease. To assess fibrosis as a functional readout for this model, lungs at terminal timepoint (14-15 dpi) were processed for flow cytometry, wherein Pdgfra^{+} mesenchymal cells were intracellularly stained for expression of smooth muscle actin (αSMA), a proxy for fibrosis induction. n=7 mice per bleomycin groups, 2 mice in control group. Graphs contain all values from 2 separate experiments. Statistical analysis for comparisons between two groups was done using two-tailed unpaired t tests, and for longitudinal analysis across all timepoints was done using two-way repeated measures ANOVA. Mean and standard error displayed on graphs. ns: not significant.

Gating strategy for splenic Treg cells (unenriched, unsorted)

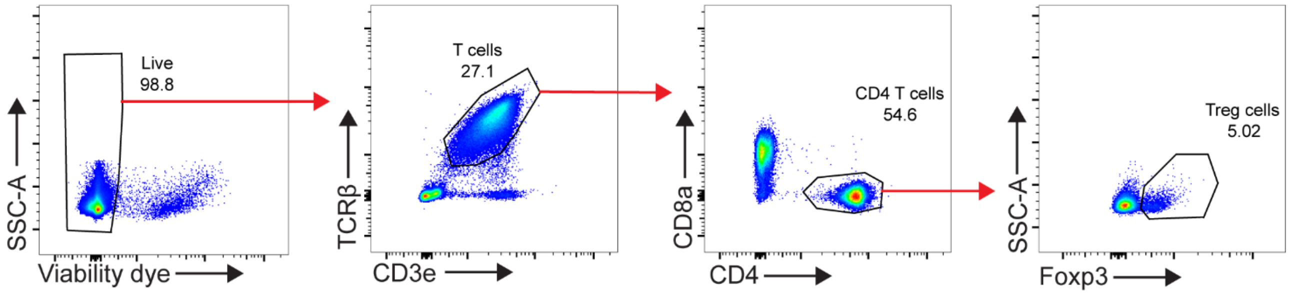

Gating strategy for spleen/lymph node Treg cell sorting (post-enrichment)

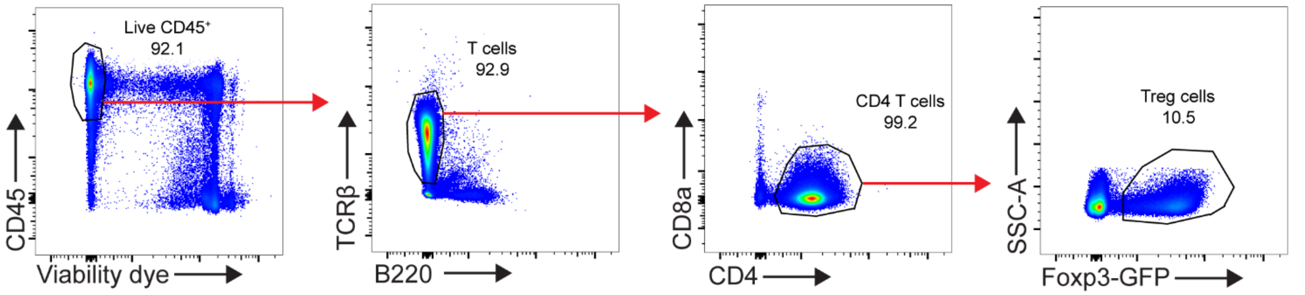

Gating strategy for lung Treg cell sorting (post-enrichment)

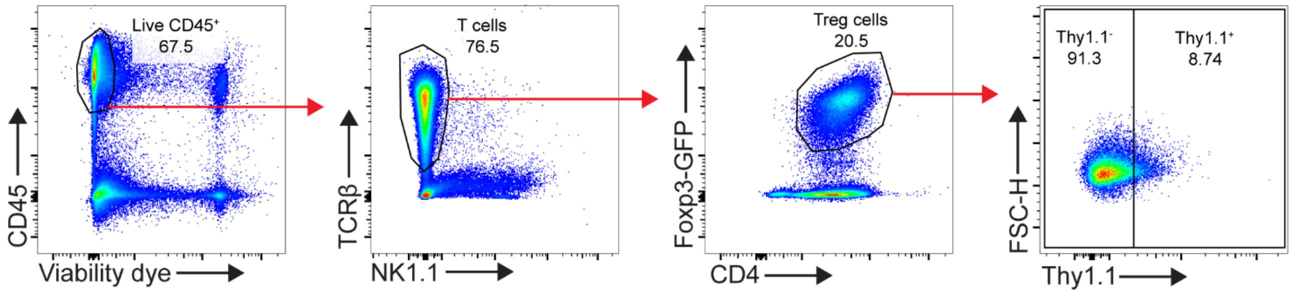

Gating strategy for *Col14a1*+ lung mesenchymal cells (Col14-LMC) sorting/co-culture assays (post-enrichment)

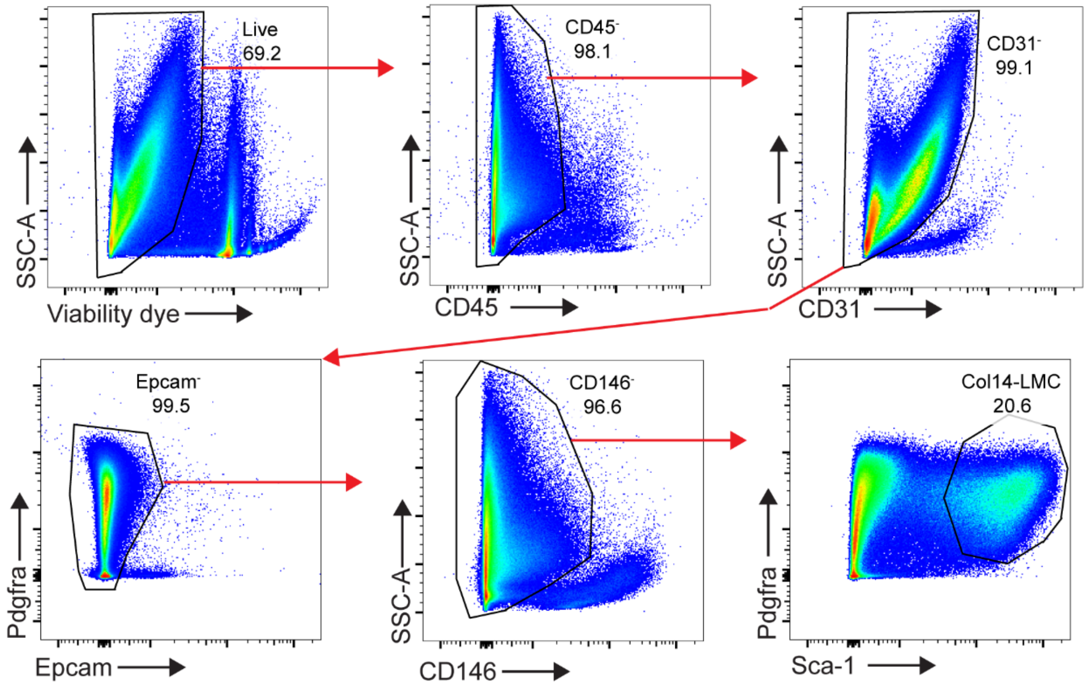

Supplemental Figure 2. Gating strategies used in experiments.

Flow cytometric gating strategies for assessment of splenic Treg cells (unenriched/unsorted), sorting of spleen/lymph node (untreated shown) or lung Treg cells (bleomycin 21 dpi shown) (post-negative enrichment for CD4 T cells, see Methods), or sorting of Col14-LMC (post-negative enrichment for mesenchymal cells, see Methods).

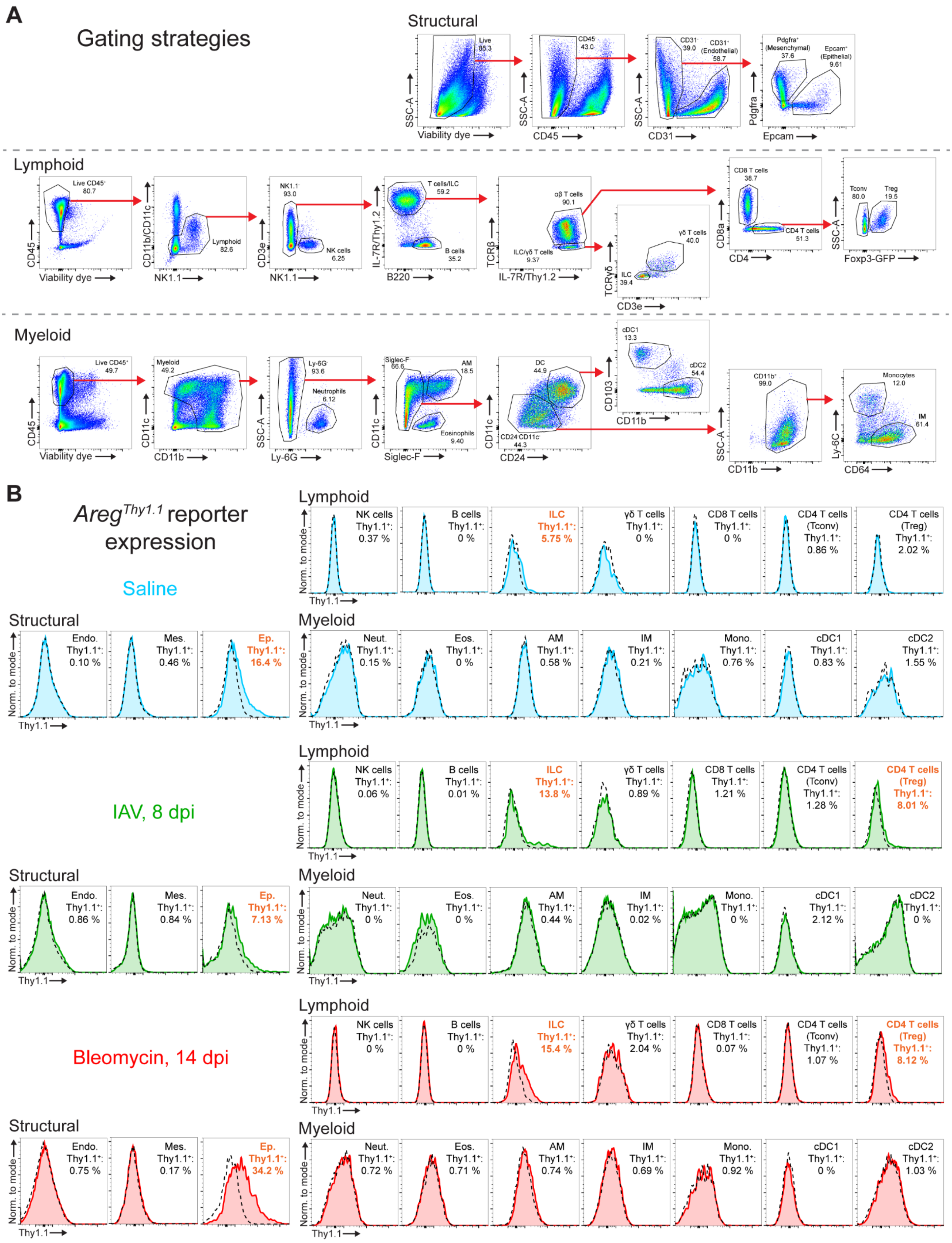
A
Gating strategies
Structural
Live 85.3
CD45- 43.0
CD31- 39.0
CD31+ (Endothelial) 58.7
Pdgfra+ (Mesenchymal) 37.6
Epcam+ (Epithelial) 9.61
SSC-A
Viability dye
CD45
CD31
Pdgfra
Epcam
Lymphoid
Live CD45+ 80.7
Lymphoid 82.6
NK1.1- 93.0
NK cells 6.25
T cells/ILC 59.2
B cells 35.2
αβ T cells 90.1
ILC/γδ T cells 9.37
γδ T cells 40.0
ILC 39.4
CD8 T cells 38.7
CD4 T cells 51.3
Tconv 80.0
Treg 19.5
CD45
Viability dye
CD11b/CD11c
NK1.1
CD3e
IL-7R/Thy1.2
B220
TCRβ
TCRγδ
CD8a
CD4
SSC-A
Foxp3-GFP
Myeloid
Live CD45+ 49.7
Myeloid 49.2
Ly-6G- 93.6
Neutrophils 6.12
Siglec-F- 66.6
AM 18.5
Eosinophils 9.40
DC 44.9
CD24- CD11c- 44.3
cDC1 13.3
cDC2 54.4
CD11b+ 99.0
Monocytes 12.0
IM 61.4
CD45
Viability dye
CD11c
CD11b
SSC-A
Ly-6G
Siglec-F
CD24
CD103
Ly-6C
CD64
B
Areg^Thy1.1 reporter expression
Saline
Lymphoid
NK cells Thy1.1+: 0.37 %
B cells Thy1.1+: 0 %
ILC Thy1.1+: 5.75 %
γδ T cells Thy1.1+: 0 %
CD8 T cells Thy1.1+: 0 %
CD4 T cells (Tconv) Thy1.1+: 0.86 %
CD4 T cells (Treg) Thy1.1+: 2.02 %
Structural
Endo. Thy1.1+: 0.10 %
Mes. Thy1.1+: 0.46 %
Ep. Thy1.1+: 16.4 %
Myeloid
Neut. Thy1.1+: 0.15 %
Eos. Thy1.1+: 0 %
AM Thy1.1+: 0.58 %
IM Thy1.1+: 0.21 %
Mono. Thy1.1+: 0.76 %
cDC1 Thy1.1+: 0.83 %
cDC2 Thy1.1+: 1.55 %
IAV, 8 dpi
Lymphoid
NK cells Thy1.1+: 0.06 %
B cells Thy1.1+: 0.01 %
ILC Thy1.1+: 13.8 %
γδ T cells Thy1.1+: 0.89 %
CD8 T cells Thy1.1+: 1.21 %
CD4 T cells (Tconv) Thy1.1+: 1.28 %
CD4 T cells (Treg) Thy1.1+: 8.01 %
Structural
Endo. Thy1.1+: 0.86 %
Mes. Thy1.1+: 0.84 %
Ep. Thy1.1+: 7.13 %
Myeloid
Neut. Thy1.1+: 0 %
Eos. Thy1.1+: 0 %
AM Thy1.1+: 0.44 %
IM Thy1.1+: 0.02 %
Mono. Thy1.1+: 0 %
cDC1 Thy1.1+: 2.12 %
cDC2 Thy1.1+: 0 %
Bleomycin, 14 dpi
Lymphoid
NK cells Thy1.1+: 0 %
B cells Thy1.1+: 0 %
ILC Thy1.1+: 15.4 %
γδ T cells Thy1.1+: 2.04 %
CD8 T cells Thy1.1+: 0.07 %
CD4 T cells (Tconv) Thy1.1+: 1.07 %
CD4 T cells (Treg) Thy1.1+: 8.12 %
Structural
Endo. Thy1.1+: 0.75 %
Mes. Thy1.1+: 0.17 %
Ep. Thy1.1+: 34.2 %
Myeloid
Neut. Thy1.1+: 0.72 %
Eos. Thy1.1+: 0.71 %
AM Thy1.1+: 0.74 %
IM Thy1.1+: 0.69 %
Mono. Thy1.1+: 0.92 %
cDC1 Thy1.1+: 0 %
cDC2 Thy1.1+: 1.03 %
Norm. to mode
Thy1.1

Supplemental Figure 3. *Areg$^{Thy1.1}$* reporter expression across lung cell populations

A) Flow cytometry gating strategies for structural, lymphoid, and myeloid cell types in the mouse lung (from bleomycin treated mouse at 14 dpi). Percent of previously gated population shown in plots. **B)** Thy1.1 staining (solid line/shaded) in populations from (A) from *Areg$^{Thy1.1}$* reporter mice in indicated models. Dotted lines represent FMO staining controls. Percentages on plots given for Thy1.1^{+} staining (with FMO staining percentage subtracted). Bold/orange text indicates percentage >5% staining. Representative staining shown from 2-4 experiments.

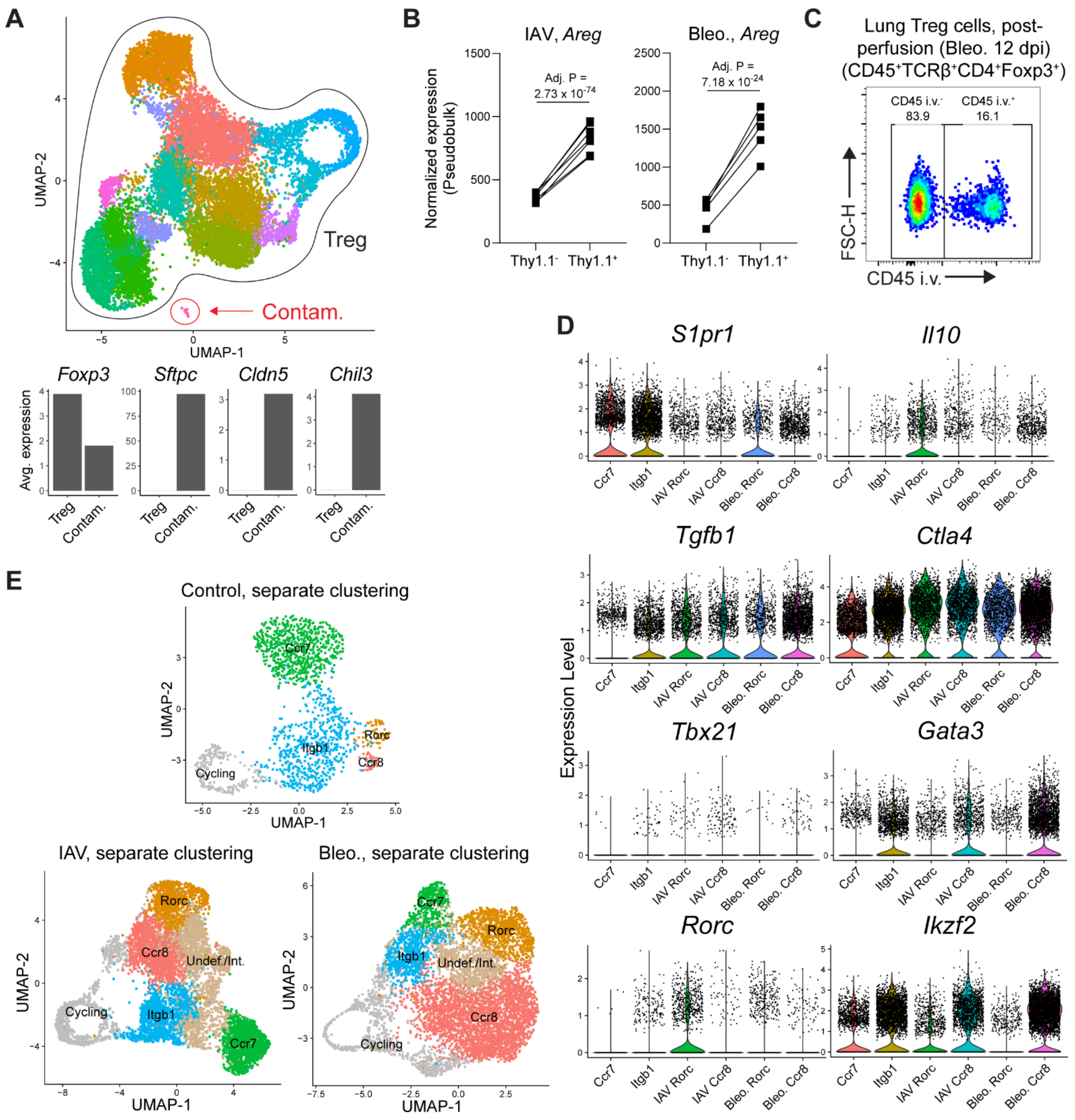

Supplemental Figure 4. Supplemental data from Figure 3.

A) Top: UMAP of clustered cells from scRNA-seq gene expression analysis using Seurat (see Fig. 3A), prior to removal of contaminating non-Treg cells ("Contam." on graph). Bottom: Graphs indicating average expression of *Foxp3* (Treg cell-specific) or Treg cell-nonspecific genes (*Sftpc*: epithelial, *Cldn5*: endothelial, *Chil3*: macrophage), in contaminating population compared to other cells in dataset ("Treg"). **B)** *Areg* expression from separate mice (counts aggregated via pseudobulk analysis and normalized/compared using DESeq2), with paired analysis of Thy1.1^{-} vs. Thy1.1^{+} Treg cells, from IAV and bleomycin datasets. Adjusted p-values displayed on graphs. **C)** Flow cytometry of Treg cells from bleomycin-treated lungs (14 dpi), treated intravenously prior to harvest with CD45 antibody ("CD45 i.v.") to identify circulating cells. Lungs had undergone an identical perfusion procedure to that used for all experiments herein. Representative staining from 3 experiments. **D)** Violin plots indicating per-cell expression of select genes in combined control/IAV/bleomycin dataset. **E)** Separate re-clustering of control, IAV, and bleomycin cells for better delineation of subgroups.

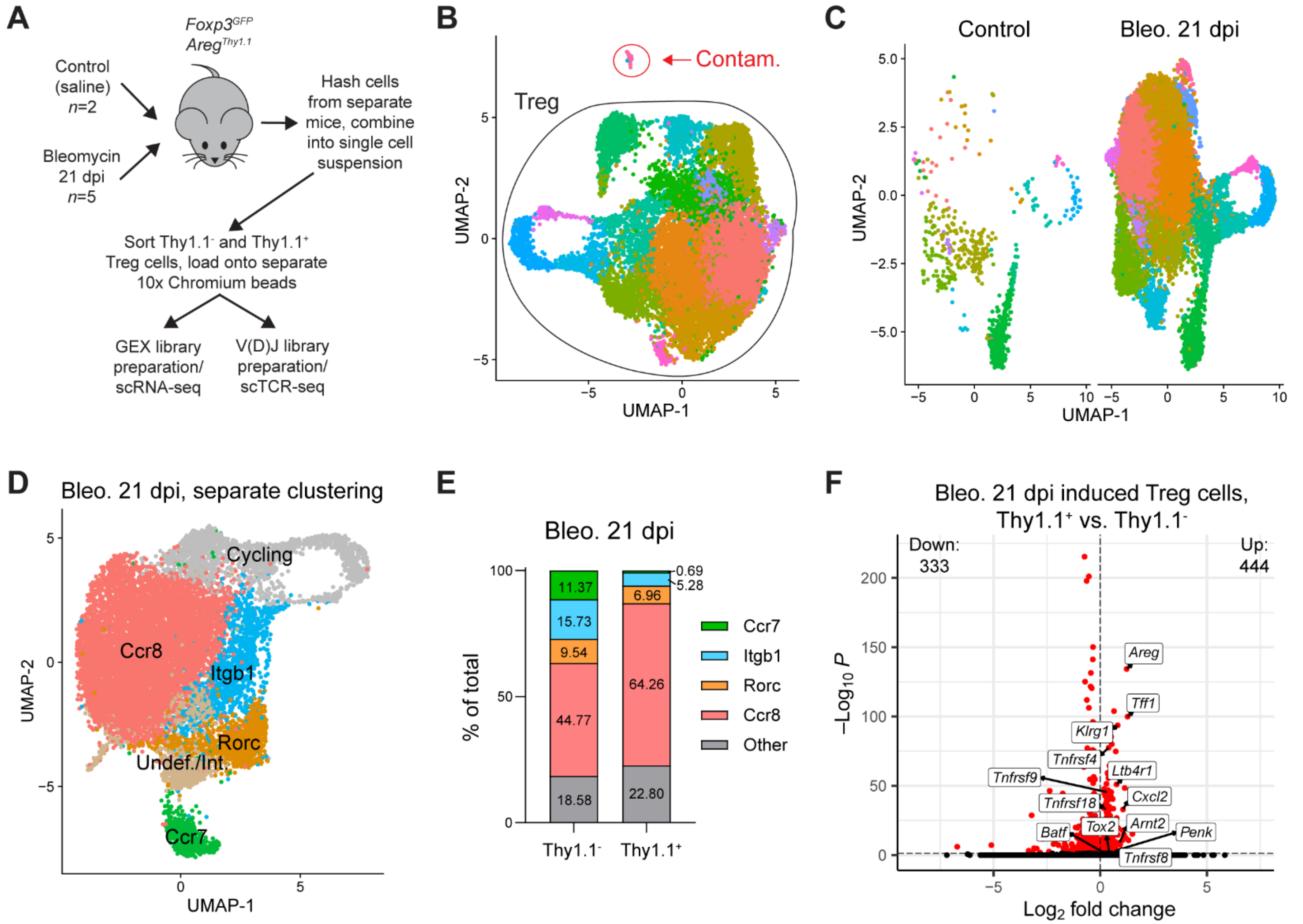

Supplemental Figure 5. scRNA-seq of Areg-producing and non-producing lung Treg cells from bleomycin-treated mice at a later timepoint.

A) Schematic of scRNA-seq experiments using lung Thy1.1$^+$ vs. Thy1.1$^-$ Treg cells from the bleomycin model at 21 dpi (as well as control, saline-treated mice). **B)** UMAP of clustered cells from gene expression analysis of the bleomycin 21 dpi dataset using Seurat, prior to removal of contaminating non-Treg cells ("Contam." on graph). **C)** UMAP of cell clustering after removal of contaminating cells, split by treatment status of Treg cells. **D)** Individual re-clustering of bleomycin 21 dpi cells for better delineation of subgroups. **E)** Proportions of each assigned subgroup in Thy1.1$^-$ and Thy1.1$^+$ Treg cells from the bleomycin 21 dpi dataset (from individual clustering). **F)** Volcano plot of DEGs from Thy1.1$^+$ vs. Thy1.1$^-$ induced Treg cells (Ccr8 and Rorc subgroups combined) from bleomycin 21 dpi dataset. Red dots on volcano plots: significant DEGs (FDR adj. p-value < 0.05). No fold change cutoff. Numbers of significantly upregulated and downregulated genes indicated on plots.

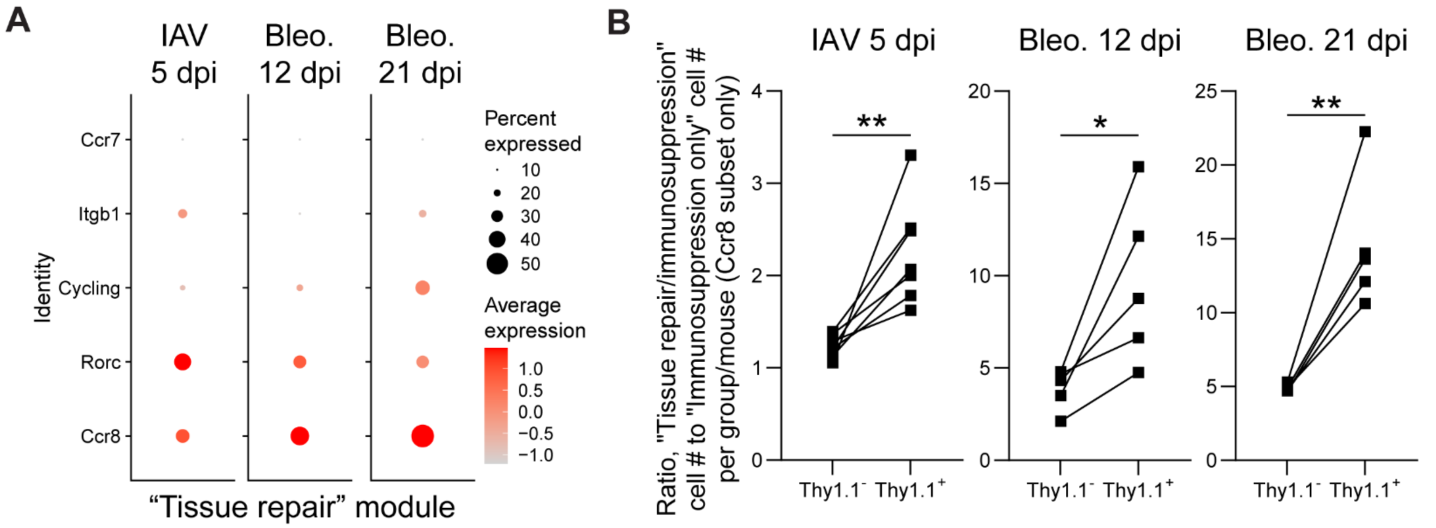

Supplemental Figure 6. Supplemental data from Figure 5.

A) Expression levels of "Tissue repair" gene module (Fig. 5A) in Treg subgroups defined by clustering, for each scRNA-seq dataset. **B)** Ratio of number of cells in the "Tissue repair/immunosuppression" group to the number of cells in the "Immunosuppression only" group, as defined by the module analysis in Fig. 5B, in Thy1.1$^-$ or Thy1.1$^+$ cells from each separate mouse in datasets (Ccr8 subgroup only). Statistical analysis for comparisons between two groups was done using two-tailed paired t tests, between Thy1.1$^-$ and Thy1.1$^+$ cells from same mice. *: 0.01<p<0.05, **: 0.001<p<0.01

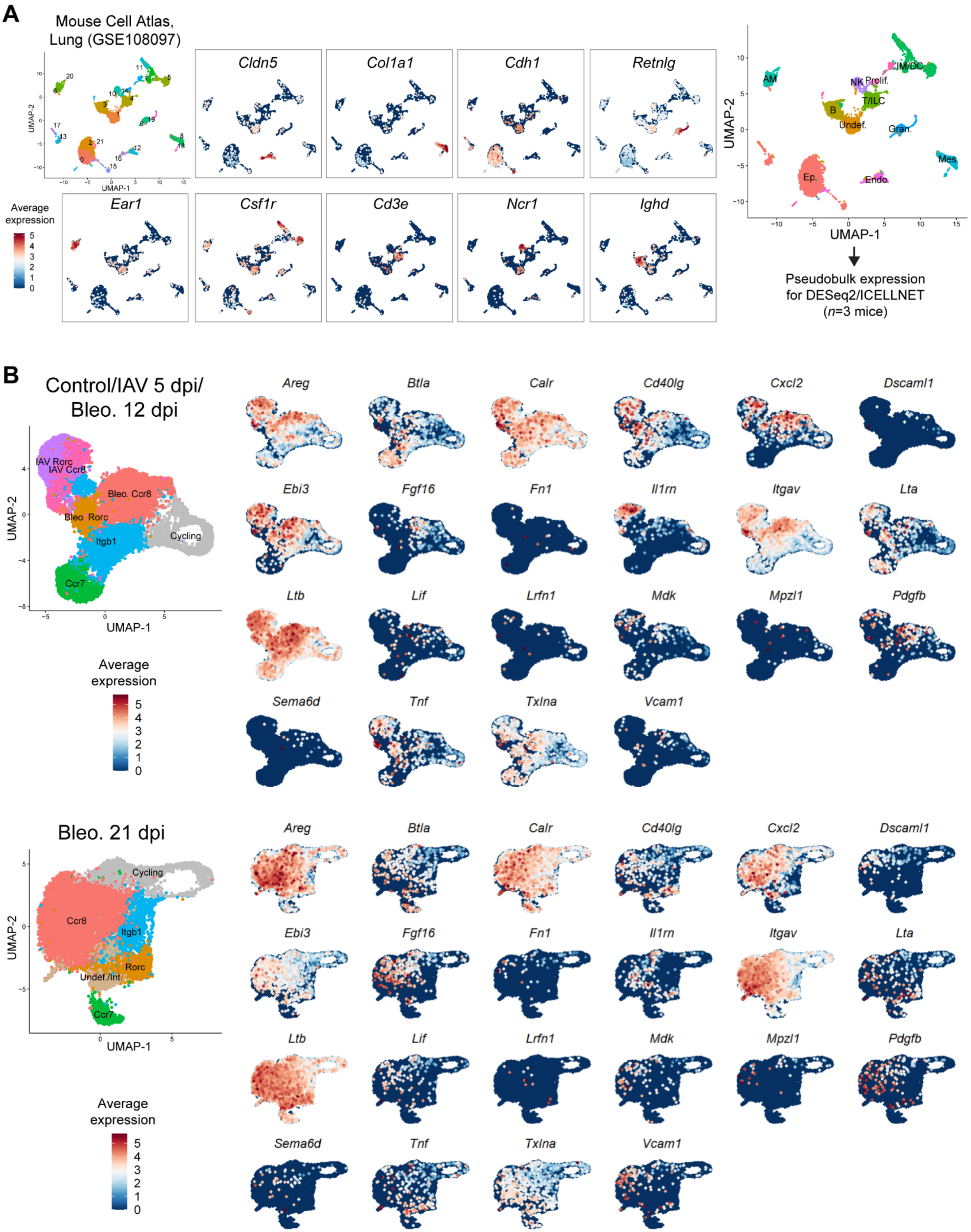
A
Mouse Cell Atlas,
Lung (GSE108097)
UMAP-1
UMAP-2
Cldn5
Col1a1
Cdh1
Retnlg
Average
expression
Ear1
Csf1r
Cd3e
Ncr1
Ighd
AM
IM/DC
NK
Prolif.
T/ILC
B
Undef.
Gran.
Mes.
Ep.
Endo.
Pseudobulk expression
for DESeq2/ICELLNET
(n=3 mice)
B
Control/IAV 5 dpi/
Bleo. 12 dpi
IAV Rorc
IAV Ccr8
Bleo. Ccr8
Bleo. Rorc
Itgb1
Cycling
Ccr7
Areg
Btla
Calr
Cd40lg
Cxcl2
Dscaml1
Ebi3
Fgf16
Fn1
Il1rn
Itgav
Lta
Ltb
Lif
Lrfn1
Mdk
Mpzl1
Pdgfb
Sema6d
Tnf
Txlna
Vcam1
Bleo. 21 dpi
Ccr8
Rorc
Undef./Int.

Supplemental Figure 7. Supplemental data for Figure 7.
A) Reanalysis of lung cells from the Mouse Cell Atlas scRNA-seq dataset (GSE108097). Endo.: endothelium; Mes.: mesenchyme; Ep.: epithelium; AM: alveolar macrophages; IM/DC: interstitial macrophages/dendritic cells; NK: NK cells; T/ILC: T cells/innate lymphoid cells; Gran.: granulocytes; B: B cells. **B)** Expression levels from scRNA-seq datasets from this study of Treg cell ligand genes identified in Fig. 7E.

Supplemental Tables (included in "Supplemental Tables" Excel file)

Supplemental Table 1. Significant DEGs from bulk RNA-seq comparison of Thy1.1$^+$ vs. Thy1.1$^-$ Treg cells from IAV-infected lungs (8 dpi) (paired analysis)

Supplemental Table 2. Significant DEGs from bulk RNA-seq comparison of Thy1.1$^+$ vs. Thy1.1$^-$ Treg cells from bleomycin-treated lungs (12 dpi) (paired analysis)

Supplemental Table 3. Significant DEGs from single cell RNA-seq comparison of IAV-induced lung Treg cells (5 dpi) (Rorc and Ccr8 groups combined) vs. bleomycin-induced lung Treg cells (12 dpi) (Rorc and Ccr8 groups combined)

Supplemental Table 4. Significant DEGs from single cell RNA-seq (IAV 5 dpi) comparison of Thy1.1$^+$ vs. Thy1.1$^-$ lung Treg cells, induced subsets only (Rorc and Ccr8 groups combined)

Supplemental Table 5. Significant DEGs from single cell RNA-seq (bleomycin 12 dpi) comparison of Thy1.1$^+$ vs. Thy1.1$^-$ lung Treg cells, induced subsets only (Rorc and Ccr8 groups combined)

Supplemental Table 6. Significant DEGs from single cell RNA-seq (bleomycin 21 dpi) comparison of Thy1.1$^+$ vs. Thy1.1$^-$ lung Treg cells, induced subsets only (Rorc and Ccr8 groups combined)

Supplemental Table 7. TCR sequences (amino acid level) for all complete (CDR3α and CDR3β) TCRs found on Treg cells in all scRNA/TCR seq datasets, with frequencies shown for each separate mouse in each dataset

Supplemental Table 8. Significant DEGs from single cell RNA-seq (bleomycin 21 dpi) comparison of Ccr8 subgroup Treg cells with no clonal expansion vs. with TCR expansion ≥ 10

Supplemental Table 9. Significant DEGs from single cell RNA-seq (IAV 5 dpi) comparison of Ccr8 subgroup Treg cells in "Tissue repair/immunosuppression" group vs. "Immunosuppression only" group from module analysis

Supplemental Table 10. Significant DEGs from single cell RNA-seq (bleomycin 12 dpi) comparison of Ccr8 subgroup Treg cells in "Tissue repair/immunosuppression" group vs. "Immunosuppression only" group from module analysis

Supplemental Table 11. Significant DEGs from single cell RNA-seq (bleomycin 21 dpi) comparison of Ccr8 subgroup Treg cells in "Tissue repair/immunosuppression" group vs. "Immunosuppression only" group from module analysis

Supplemental Table 12. Significant DEGs from bulk RNA-seq comparison of 4-1BB activating antibody (3H3) ex vivo stimulated (4h) vs. IgG control Treg cells from bleomycin-treated lungs (15 dpi) (paired analysis)

Supplemental Methods

Mice

Areg$^{Thy1.1/Thy1.1}$ mice were a novel creation for these studies. To create this strain, we inserted a P2A self-cleaving viral peptide followed by a mouse *Thy1*^{a} (*Thy1.1*) sequence into the endogenous mouse *Areg* locus prior to the native stop codon with a subsequent FRT-flanked neomycin resistance cassette. Following recombineering of this construct and transduction, mouse embryonic stem cells positive for this construct were isolated by neomycin treatment, then microinjected into mouse embryos to create heterozygotes (on the C57BL6/N *Thy1*^{b} [*Thy1.2*] background), and the FRT-flanked neomycin cassette was then removed by crossing with the FLPeR mouse. We then homozygosed these mice by breeding to create *Areg*$^{Thy1.1/Thy1.1}$ mice (referred to as *Areg*$^{Thy1.1}$ mice in the report). *Foxp3*^{GFP} mice were a generous gift from the laboratory of Dr. Alexander Rudensky (Memorial Sloan Kettering, New York, NY, USA), and were previously described (Fontenot *et al.* 2005). Wild type (WT) mice (C57BL/6N) were acquired and bred from Jackson Laboratory stocks (Strain #:005304). These mice or lab-bred descendants were utilized for Col14-LMC isolation/sorting.

Bulk RNA-seq

For bulk RNA-seq of Thy1.1^{-} vs. Thy1.1^{+} lung Treg cells, lungs from 4 *Foxp3*^{GFP}*Areg*$^{Thy1.1}$ IAV-treated mice (275 TCID50 PR8/H1N1) (8 dpi) and 3 *Foxp3*^{GFP}*Areg*$^{Thy1.1}$ bleomycin-treated mice (1 U/kg) (12 dpi) were harvested, enriched for CD4 T cells, prepared for flow cytometry, and sorted (Treg cells) as described in relevant later sections. 5,000 Thy1.1^{-} and 5,000 Thy1.1^{+} Treg cells were sorted per sample. Following sorting, samples were centrifuged at 550 x g/8 min./4°C, supernatants were aspirated, and samples flash frozen. RNA was extracted, cDNA libraries were generated, and sequencing was performed at 40 million reads/sample by Genewiz from Azenta Life Sciences. For bulk RNA-seq of lung Treg cells with 4-1BB agonistic antibody stimulation, lungs from 10 *Foxp3*^{GFP}*Areg*$^{Thy1.1}$ bleomycin-treated mice (1 U/kg) (15 dpi) were harvested, enriched for CD4 T cells, prepared for flow cytometry, and sorted (Treg cells) as described in relevant later sections. Treg cells were pooled from

2-4 mice to obtain 100,000-120,000 cells for each pool, which post-sorting were each split into 2 matched wells in a 48 well tissue culture-treated plates (Corning), with 50,000-60,000 cells/well (8 wells total) in complete T cell media (RPMI + 100x penicillin/streptomycin, 100x GlutaMAX, 100x HEPES, 100x sodium pyruvate, 100x nonessential amino acids, 1000x β-mercaptoethanol [all Gibco], and 10% fetal bovine serum [FBS]) with rhIL-2 (200 U/ml; NCI Preclinical Repository) and rhIL-7 (10 ng/ml; NCI Preclinical Repository). Wells were then treated with InVivoMAB anti-mouse 4-1BB agonistic antibody (clone 3H3; BioXCell, BE0239) (10 μg/ml) or InVivoMAb rat IgG2a isotype control, anti-trinitrophenol (clone 2A3; BioXCell, BE0089) (10 μg/ml). After 4h of incubation, Treg cells were washed out of wells, centrifuged at 550 x g/8 min., supernatant was aspirated, then cells were lysed in Trizol Reagent (Thermo). RNA was extracted by the Columbia Molecular Pathology Shared Resource using the miRNeasy Micro Kit (Qiagen), and RINs were found to be >9.8 via Bioanalyzer analysis. RNA-seq was performed at the Columbia Genome Center, using SMART-Seq v4 Ultra Low Input RNA Kit for Sequencing (Takara) for reverse transcription and RNA amplification of small RNA amounts, with subsequent library preparation with a Nextera XT Kit (Illumina) and sequencing with a NovaSeq 6000 sequencer (Illumina) at 40 million reads/sample. Base calling was done with RTA (Illumina) and bclfastq2 (version 2.19) was used to convert BCL to fastq files. For both bulk RNA-seq datasets, fastq files were aligned using RNA Detector (La Ferlita *et al.* 2021), with adapter trimming using Trim Galore and pseudoalignment using Salmon to generate RNA counts. DESeq2 (in the R interface) (Love *et al.* 2014) was used for analysis (paired analysis between Thy1.1$^-$ vs. Thy1.1$^+$ Treg cells from each mouse, or between IgG control vs. 4-1BB agonistic antibody treated cells from paired Treg cell pools). Counts were filtered to only include genes with ≥10 counts total across all samples. ComplexHeatmap (Gu *et al.* 2016) and EnhancedVolcano R packages were used for analysis, and GSEA (Subramanian *et al.* 2005) was used for pathway analysis. R was used for Venn diagram analysis. ICELLNET (version 2) was used for receptor-ligand analyses (Noël *et al.* 2021, Massenet-Regad *et al.* 2024), with normalized expression values from DESeq2. All genes were used for total cell interaction scores, while only significant DEGs from the IgG control vs. 4-1BB agonistic antibody comparison were used to probe

individual ligand/receptor interactions with mesenchyme, epithelium, and endothelium. Interaction scores were computed by taking the product of the normalized expression levels of ligand on the central cell and receptor on the partner cell (then summed for total interaction score). Secondary analysis of data from the Mouse Cell Atlas, lung dataset (Han *et al.* 2018) (GSE108097) was done using Seurat (Stuart *et al.* 2019), pseudobulked by mouse for separate cell types, and normalized using DESeq2 for use with ICELLNET.

<u>*Single cell RNA- and TCR-seq*</u>

For the first experiment, 7 IAV-treated mice (275 TCID50 PR8/H1N1) (5 dpi), 5 bleomycin-treated mice (1 U/kg) (12 dpi), and 2 control mice (saline-treated, 5 dpi) were used; for the second experiment, 5 bleomycin-treated mice (1 U/kg) (21 dpi), and 2 control mice (saline-treated, 5 dpi) were used. All mice were *Foxp3*^{GFP}*Areg*$^{Thy1.1}$. Lungs were harvested, enriched for CD4 T cells, and prepared for flow cytometry as described in relevant later sections. Hashing was performed on cells from separate mice with custom αCD2 hashes (10 min. of FC block incubation prior to staining, 0.5 ug of hash per 2 million cells for 30 min. at 4°C); custom hashes were conjugated as described in protocols of the Technology Innovation Lab at the New York Genome Center using iEDDA-click chemistry. Afterwards, cells from separate mice were combined, stained for sorting antibodies, and sorted as described in relevant later sections. Cells amounts to sort were determined based on optimal protocols for 10x Chromium Beads (~1000-3000 cells per mouse; ~15,000 total cells per bead). Thy1.1$^-$ and Thy1.1$^+$ cells were run on separate beads in parallel to eliminate the need for a separate hashing step post-sorting. Thy1.1$^-$ and Thy1.1$^+$ Treg cells were included in roughly equal amounts, to give us the ability to better determine heterogeneity and representation within the Thy1.1$^+$ group. Control mouse Treg cells were all Thy1.1$^-$. Sorted Treg cells were loaded as described by the manufacturer protocols onto 10x Chromium Genomic V1 Platform (10x Genomics) and libraries were prepared for single cell 5' transcripts, TCRαβ transcripts, and CITE-seq hash libraries (Chromium Single Cell V(D)J Reagent Kit). SPRIselect Beads (BD) were used in the preparation process. Cell Ranger software (version 5.0.0) (10x Genomics) was

used to process reads (gene expression, CITE-seq, TCRαβ), with raw base call files demultiplexed with cellranger mkfastq function. This was subsequently aligned to the mm10 reference genome, then unique molecular identifiers (UMI) were collapsed to count matrices using cellranger multi function. Raw hash reads were processed for analysis with CITE-seq-Count (GitHub), in order to create a hash count matrix. Hashes were then normalized by total counts across cells, with total UMI counts set to 100, with identity assigned to the most highly expressed hashtag that represented a minimum of 2/3 of the recovered mapped UMIs in a cell. Cells with suboptimal UMI total counts (UMI total < 5 or UMI total > 5) were removed from downstream analyses. The Seurat package (in R) (Stuart *et al.* 2019) was used for analysis of single cell datasets. Cells were subsetted to exclude cells with < 200 or > 3500 features or a mitochondrial percent of features > 5%. Top 2000 top variable features were used to determine clustering (with TCR genes excluded). Since Thy1.1$^-$ and Thy1.1$^+$ cells were run on separate beads, Canonical Correlation Analysis (CCA) was performed to adjust for any batch effects between samples. 20-25 principal components were used to generate UMAPs, with clustering resolution set from 0.7-1.9 for different datasets. “Pseudobulking” of expression values for each mouse and subsequent analysis in DEseq2 (Love *et al.* 2014) was used for certain analyses. EnhancedVolcano R package was used for analysis, and GSEA (Subramanian *et al.* 2005) was used for pathway analysis. For TCR analysis, all data was generated with R using the Immunarch package (Nazarov *et al.* 2023) (for Chao1 analysis), ggPlot2 package, UpsetR package (Lex *et al.* 2014), and EnhancedVolcano package. P_{gen} was calculated using Python with code provided by the Optimized Likelihood estimate of immunoGlobulin Amino-acid sequences (OLGA) framework (Sethna *et al.* 2019).

Mouse lung damage models and assessment

Influenza A (IAV) virus (PR8/H1N1) was a generous gift from the laboratory of Dr. Donna Farber (Columbia University, New York, NY, USA). For IAV infection, mice were given ketamine/xylazine for anesthesia, then infected intranasally with 100-300 TCID50 of virus diluted in 1x PBS (as determined by the Farber lab using Madin-Darby canine kidney epithelial cell infection assays). 10-16 week old

male mice were used for IAV experiments, and mouse lungs were harvested at 5 or 8 days post-inoculation (dpi). Bleomycin (Sigma or Teva) was diluted in sterile 0.9% saline (0.5 U/ml). Bleomycin was administered to mice using one of two methods for different experiments: surgical intratracheal or oropharyngeal intratracheal administration. For surgical intratracheal administration, we followed a previously described technique (Orlando *et al.* 2019). Briefly, mice were given buprenorphine prior to experimentation for analgesia, then ketamine/xylazine for anesthesia. Once unconscious and unresponsive to toe pinch, the area of surgery was cleaned using 3 alternating scrubs of PVP Iodine Prep Pads (Medline) and WEBCOL Alcohol Preps (Covidien). A scalpel was then used to make an incision in the center of the neck, then the salivary glands were split and the muscles overlaying the trachea were carefully cut to expose the trachea. An insulin syringe (Corning) was loaded with 30-50 µl of bleomycin (calculated for 1 U/kg administration), with air pockets above and below liquid to distribute liquid into lungs. The syringe was inserted into the trachea and plunged to aspirate the solution. The surgical incision was sealed with Vetbond Tissue Adhesive (3M) and reinforced with Reflex 9mm Wound Clips (Roboz Surgical Instrument). Buprenorphine was administered 3 times at 12h intervals following surgery for analgesia, and mice were monitored for 10 days to ascertain wound closure and healing. For oropharyngeal intratracheal administration, 50 µl per mouse was administered oropharyngeally (~1 U/kg), following a previously described technique (De Vooght *et al.* 2009). Briefly, mice were given ketamine/xylazine for anesthesia, then placed on an apparatus suspending them at a 60° angle from horizontal by surgical suture string from their teeth. The tongue was removed from the mouth and held with padded forceps, then the bleomycin solution was pipetted into the back of the mouth, followed immediately by plugging the nose with padded forceps to induce oral inhalation of the solution. 10-16 week old male mice were used for bleomycin experiments, and mouse lungs were harvested at 11-21 dpi. Littermate, age-matched mice were used for all experiments. For IAV and bleomycin experiments, mice were weighed every 1-4 days. In certain experiments, mouse body temperature was assessed with a rectal thermometer every 1-4 days. For IAV experiments, mouse blood oxygen saturation (SpO_2) was assessed using a MouseOx Plus Pulse Oximeter (Starr Life

Sciences). The area around mouse the mouse neck was shaved and Nair Hair Remover Product was applied to chemically remove residual hair at the time of IAV infection. A Small Mouse Collar Sensor (Starr Life Sciences) was used to assess SpO_2 on unanesthetized at indicated timepoints during IAV infection progression, with mice placed in a 1 L beaker to restrict movement; assessment was taken for 2-5 min. per mouse at each timepoint, with only high-quality, error-free readings taken and averaged to determine a composite SpO_2 value. For histology of various organs, lung, colon, skin, liver, muscle, heart, kidney, and brain were isolated from untreated mice without perfusion, fixed in 10% neutral buffered formalin (Epredia), then sent to Histology Consultation Services for embedding in paraffin, sectioning at 5 µm, and H&E staining; full organs were imaged using a Aperio AT2 (Leica) full slide scanner at Columbia University's Molecular Pathology Shared Resource (MPSR), and representative images were taken with ImageScope software (Aperio).

Lung, spleen, and lymph node processing

For lung processing, mice were euthanized and dissected to expose the lungs. Perfusion of the lungs was performed, after nicking the left femoral artery and the left atrium of the heart, through the left ventricle of the heart with 10 ml of cold 1x PBS. Lungs were excised and placed in 0.5 ml tissue preparation media (RPMI with 100x penicillin/streptomycin, 100x GlutaMAX, 100x HEPES [all Gibco], and 5% FBS [Corning]) in a 5 ml Eppendorf tube, where they were minced. 3.5 ml was added to tubes of tissue preparation media with 5 U/ml DNAse, 1 mg/ml of collagenase A, and 1 mg/ml of dispase. (Dispase was pre-dissolved in 1x PBS consisting of 10% of the volume of the final digestion mixture.) Lungs were digested in shaking incubator set to 110 rpm at 37°C for 1h. Suspensions were then poured over a 100 µm cell strainer (Corning) into a 50 ml Falcon tube (Corning), pushed through the mesh with the top of a syringe, rinsed with 10 ml tissue preparation media, pushed through again, then rinsed with 5 ml tissue preparation media. Cells were centrifuged at 450 x g/4°C/5 min., then supernatants were poured off (due to delicate nature of pellet resulting from use of dispase). Pellets were resuspended in 2 ml of 1x ACK lysis buffer (deionized water with 154 mM ammonium chloride [Fisher], 10 mM

potassium bicarbonate [Fisher], and 0.1 mM EDTA disodium salt dihydrate [Fisher], pH 7.2) and incubated at room temperature for 2 min., then quenched with 10 ml tissue preparation media and ran through a 100 µm nylon mesh sheet into a 15 ml Falcon tube (Corning). Cells were centrifuged at 450 x g/4°C/5 min., the supernatant was aspirated, and cells were resuspended in 1 ml of tissue preparation media and placed on ice. Cells were then used for bead enrichment/sorting or antibody staining for flow cytometry. For spleen and lymph node processing, mice were euthanized and dissected to extract the spleen (and in some experiments, inguinal and cervical lymph nodes as well), which were excised and placed on top of a 100 µm cell strainer (Corning) in 5 ml of tissue preparation media in a 6 cm dish (Corning), pushed through the mesh with the top of a syringe, rinsed with 5 ml tissue preparation media, pushed through again, then rinsed with 5 ml tissue preparation media; contents of the dish were placed in a 15 ml Falcon tube (Corning). Cells were centrifuged at 450 x g/4°C/5 min., then supernatants were aspirated. Cells were then resuspended in 2 ml of 1x ACK lysis buffer and incubated at room temperature for 2 min., then quenched with 10 ml tissue preparation media and ran through a 100 µm nylon mesh sheet into a 15 ml Falcon tube (Corning). Cells were centrifuged at 450 x g/4°C/5 min., the supernatant was aspirated, and cells were resuspended in 1 ml of tissue preparation media and placed on ice. Cells were then used for bead enrichment/sorting, or directly in short-term stimulation experiments.

Splenic/lymph node Treg cell stimulation protocols

For the short-term stimulation protocol, *Areg*$^{Thy1.1}$ mouse splenocytes were isolated as described in "Lung, spleen, and lymph node processing", then cultured (bulk splenocytes, unsorted) for 3h in complete T cell media (RPMI + 100x penicillin/streptomycin, 100x GlutaMAX, 100x HEPES, 100x sodium pyruvate, 100x nonessential amino acids, 1000x β-mercaptoethanol [all Gibco], and 10% fetal bovine serum [FBS]) with phorbol 12-myristate 13-acetate (PMA) (50 ng/ml) (Sigma) and ionomycin (500 ng/ml) (Sigma), or unstimulated; cells were then stained for flow cytometry to identify Treg cells/Thy1.1 expression. Cells were stained for AREG production using the endogenous AREG

antibody (see "Flow cytometry"). For the long-term stimulation protocol, *Foxp3*^{GFP} and *Foxp3*^{GFP}*Areg*$^{Thy1.1}$ mouse spleen and lymph node T cells were isolated as described in "Lung, spleen, and lymph node processing", then negatively enriched for CD4 T cells and sorted for Treg cells as described in "CD4 T cell bead enrichment and Treg cell sorting". Treg cells were then cultured in complete T cell media for 96h in round bottom plates in the presence of rhIL-2 (200 U/ml; NCI Preclinical Repository) and rhIL-7 (10 ng/ml; NCI Preclinical Repository), with or without Dynabeads Mouse T-Activator CD3/CD28 for T-Cell Expansion and Activation (Thermo) beads added at a 1:1 Treg cell/bead ratio. Cells were then counted using 123count eBeads Counting Beads (Invitrogen) on a BD LSRII. Cells were stained for AREG production using the endogenous AREG antibody (see "Flow cytometry").

CD4 T cell bead enrichment and Treg cell sorting

Lungs, spleens, and/or lymph nodes were prepared as described in "Lung, spleen, and lymph node processing". Lung, spleen, and/or lymph node cells were then enriched for CD4 T cells using either positive enrichment with the Dynabeads FlowComp Mouse CD4 kit, or negative enrichment with iMag Streptavidin Particles Plus – DM beads (BD), according to their respective protocols. For negative enrichment, following a 10 min. incubation with FC block (purified anti-mouse CD16/CD32, clone 2.4G2; Cytek, 70-0161), cells were incubated in a cocktail of biotinylated antibodies towards mouse TER-119 (clone TER-119; Biolegend, 116204), CD31 (clone 390; Biolegend, 102404), Epcam (clone G8.8; Biolegend, 118204), Pdgfra (clone APA5; Biolegend, 135910), CD19 (clone 6D5; Biolegend, 115504), NK1.1 (clone PK136; Biolegend, 108704), CD11b (clone M1/70; Biolegend, 101204), CD11c (clone N418; Biolegend, 117304), and CD8a (clone 53-6.7; Biolegend, 100704) (only TER-119, CD19, NK1.1, CD11b, CD11c, and CD8a antibodies were used for spleen/lymph node preparations). Post-bead enrichment, cells were stained with fluorescent mouse antibodies for flow cytometric sorting (100 µl per mouse). Antibodies used were: CD45-BUV395 (clone 30-F11; BD, 564279), CD4-BV650 (clone RM4-5; Biolegend, 100555) or CD4-APC-Cy7 (clone RM4-5; Cytek, 25-0042), TCR β-BV711 (clone H57-597; BD, 563135) or TCR β-PE/Dazzle594 (clone H57-597; Biolegend, 109240), CD45R (B220)-

PerCP-Cy5.5 (clone RA3-6B2; Cytek, 65-0452), CD8a-PE (clone 53-6.7; Cytek, 50-0081), NK1.1-PE-Cy7 (clone PK136; Biolegend, 108714), CD90.1 (Thy1.1)-APC (clone HIS51; Invitrogen, 17090082). Treg cell preparations were done with lungs of *Foxp3*^{GFP} mice (i.e., Treg cells express GFP), allowing sorting for GFP^{+} cells. Cells were incubated with antibodies for 20 min. at 4°C. Post-staining, cells were washed, then resuspended in 200 µl per mouse of flow cytometry buffer without sodium azide, ran through a 40 µm mesh, then sorted on a BD Aria sorter, with Sytox Blue (Invitrogen) added 5 min. prior to running samples for dead cell exclusion.

Flow cytometry

Lungs were prepared for flow cytometry as described above, with 2-3 million cells from final single cell suspensions used for antibody staining and 100,000-1 million cells ran on a BD LSRII or BD Fortessa. Cells were stained in flow cytometry buffer (1x PBS with 1% BSA [Gold Biotechnology], 2.5 mM EDTA disodium salt dihydrate [Fisher], and 0.1% sodium azide [Fisher]). Zombie Violet Fixable Viability Kit (Biolegend) or GhostDye Red 780 (Cytek), stained in a separate step from surface antibodies in 1x PBS, or Sytox Blue (Thermo), added directly to sample 5 min. prior to running on cytometer, were used for dead cell exclusion. Staining was preceded by a 10 min. incubation with FC block (purified anti-mouse CD16/CD32, clone 2.4G2; Cytek, 70-0161) in flow cytometry buffer, with 2x antibody cocktail added. For analysis of spleen Treg cells from *Areg*$^{Thy1.1}$ mice, surface staining was done using these anti-mouse antibodies: CD4-BV605 (clone RM4-5; BD, 563151), CD8a-PerCP-Cy5.5 (clone 53-6.7; Cytek, 65-0081), TCR β-BV711 (clone H57-597; BD, 563135), CD3e-PE-Cy7 (clone 145-2C11, Cytek, 60-0031), CD90.1 (Thy1.1)-APC (clone HIS51; Invitrogen, 17090082) (intracellular staining for Foxp3 and AREG used, see later); in these experiments, the APC Mouse IgG2a κ Isotype Ctrl Antibody (clone MOPC-173; Biolegend, 400219) was used as a control for Thy1.1 staining. For analysis of structural cells from live lung single cell suspensions from *Foxp3*^{GFP}*Areg*$^{Thy1.1}$ mice, surface staining was done using these anti-mouse antibodies: CD45-BUV395 (clone 30-F11; BD, 564279), CD31-BV711 (clone 390; Biolegend, 102449), Epcam-BV785 (clone G8.8; Biolegend, 118245), Pdgfra-BV605 (clone APA5;

Biolegend, 135916), CD90.1 (Thy1.1)-APC (clone HIS51; Invitrogen, 17090082). For analysis of lymphoid cells from live lung single cell suspensions from *Foxp3^{GFP}Areg$^{Thy1.1}$* mice, surface staining was done using these anti-mouse antibodies: NK1.1-BUV395 (clone PK136; BD, 564144), CD3e-BUV496 (clone 145-2C11; BD, 612955), CD4-BUV737 (clone RM4-5; BD, 612843), TCR γ/δ-BV421 (clone GL3; Biolegend, 118120), CD11b-BV510 (clone M1/70; BD, 562950), CD11c-BV510 (clone HL3; BD, 562949), CD45-BV786 (clone 30-F11; BD, 564225), CD45R (B220)-PerCP-Cy5.5 (clone RA3-6B2; Cytek, 65-0452), CD8a-PE (clone 53-6.7; Cytek, 50-0081), TCR β-PE/Dazzle594 (clone H57-597; Biolegend, 109240), CD90.2 (Thy1.2)-PE-Cy7 (53-2.1; Biolegend, 140310), CD127 (IL-7Ra)-PE-Cy7 (A7R34; Cytek, 60-1271), CD90.1 (Thy1.1)-APC (clone HIS51; Invitrogen, 17090082) (*Foxp3^{GFP}* used for identifying Treg cells). For analysis of myeloid cells from live lung single cell suspensions from *Foxp3^{GFP}Areg$^{Thy1.1}$* mice, surface staining was done using these anti-mouse antibodies: CD45-BUV395 (clone 30-F11; BD, 564279), CD24-BV510 (clone M1/69; Biolegend, 101831), CD11b-BV650 (clone M1/70; Biolegend, 101259), CD103-BV711 (clone M290; BD, 564320), CD11c-FITC (clone N418; Cytek, 35-0114), Ly6C-PerCP-Cy5.5 (clone HK1.4; Biolegend, 128012), Siglec-F-PE (clone E50-2440; BD, 552126), Ly6G-PE/Dazzle594 (clone 1A8; Biolegend, 127648), CD64-PE-Cy7 (clone X54-5/7.1; Biolegend, 139314), CD90.1 (Thy1.1)-APC (clone HIS51; Invitrogen, 17090082). Following surface marker staining, for staining with anti-mouse Foxp3-FITC (clone FJK-16s; Invitrogen, 11577382), anti-mouse α-smooth muscle actin-eFluor660 (clone 1A4; Invitrogen, 50976082), or biotinylated anti-mouse AREG (endogenous) (polyclonal goat IgG; R&D systems, BAF989) followed by Streptavidin conjugates, cells were fixed/permeabilized with Foxp3/Transcription Factor Staining Buffer Kit (Cytek). For endogenous AREG staining, following staining with biotinylated anti-mouse AREG, a separate staining step with Streptavidin-PE-eFluor610 (Invitrogen) or Streptavidin-PE (Thermo) was utilized. For candidate receptor analysis Treg cells from bleomycin-treated mouse lungs (live cells used for staining), Treg cells from *Foxp3^{GFP}Areg$^{Thy1.1}$* mice were stained for as above, and proteins were assessed with flow cytometry using these antibodies: anti-mouse OX-40-PE (clone OX-86; Biolegend, 119409), anti-mouse 4-1BB-PE (clone 17B5; Biolegend, 106105), anti-mouse CD51-PE (clone RMV-7; Biolegend,

104105), anti-human/mouse LTB4R1 (clone 7A8; Sigma, MABF2769), and CD90.1 (Thy1.1)-BUV737 (clone HIS51; BD, 612837). Since anti-mouse/human LTB4R1 is an unconjugated mouse monoclonal antibody, the Mouse-on-Mouse Immunodetection Kit (Vector Laboratories) was used (including biotinylated anti-mouse IgG), with subsequent staining using Streptavidin-PE (Thermo); since intravenous CD45 injection was used in these experiments, all CD45 intravenous positive cells stain positive regardless of LTB4R1 expression due to Mouse-on-Mouse staining protocol, and thus were excluded from analysis. Fluorescence-minus-one (FMO) controls were included to define staining boundaries where necessary. For certain experiments, CD45-APC (clone 30-F11; Cytek, 20-0451) was injected intravenously into mice 5 min. prior to euthanasia, to label circulating immune cells.

Col14-LMC negative enrichment and sorting

Lungs were prepared as described in "Lung processing". Lung cells were then negatively enriched for mesenchymal cells using iMag Streptavidin Particles Plus – DM beads (BD), according to their protocol. For negative enrichment, following a 10 min. incubation with FC block (purified anti-mouse CD16/CD32, clone 2.4G2; Cytek, 70-0161), cells were incubated in a cocktail of biotinylated antibodies towards mouse CD45 (clone 30-F11; Biolegend, 103104), CD31 (clone 390; Biolegend, 102404), Epcam (clone G8.8; Biolegend, 118204), and TER-119 (clone TER-119; Biolegend, 116204). Post-bead enrichment, cells were stained with fluorescent mouse antibodies for flow cytometric sorting (100 µl per mouse); antibodies used were: CD31-BV605 (clone 390; Biolegend, 102427), Epcam-PerCP-Cy5.5 (clone G8.8; Biolegend, 118220); Pdgfra-PE (clone APA5; Biolegend, 135905), CD146-PE-Cy7 (clone ME-9F1; Biolegend, 134714), CD45-APC (clone 30-F11; Cytek, 20-0451), and Sca-1-APC-Cy7 (clone D7; Biolegend, 108126). Post-staining, cells were washed, then resuspended in 200 µl per mouse, ran through a 70 µm mesh, then sorted on a BD Aria sorter, with Sytox Blue added 5 min. prior to running samples for dead cell exclusion.

Col14-LMC/Treg cell co-culture

Col14-LMC were isolated/sorted as described and plated in mesenchymal cell media at 40,000-50,000 cells/well in 48 well tissue culture-treated plates (Corning). Cells were allowed to adhere overnight (16-18h); the following day, media was aspirated from cells, cells were washed with RPMI (Gibco) to remove dead cells, and fresh T cell media was added; cells were rested for ~12h while Treg cell isolation/sorting occurred (~8h). Treg cells from lungs or spleens of bleomycin-treated mice (11-15 dpi) were isolated/sorted as described above. 20,000-25,000 Treg cells (1:2 Treg cell:Col14-LMC ratio) were then added directly to Col14-LMC wells in small volumes (1/25 volume of media in wells). Dynabeads Mouse T-Activator CD3/CD28 for T-Cell Expansion and Activation (Thermo) beads were added to Treg cells at a 1:1 Treg cell/bead ratio prior to addition to wells (in all assays except where otherwise indicated). rhIL-2 (200 U/ml; NCI Preclinical Repository) and rhIL-7 (10 ng/ml; NCI Preclinical Repository) were added directly to wells at time of Treg cell addition. For some experiments, anti-mouse AREG antibody (polyclonal goat IgG; R&D Systems, AF989) (1 μg/ml) or normal goat IgG (R&D Systems, AB108C) (1 μg/ml) were added to co-cultures. For some experiments, Treg cells were separated from Col14-LMC with a 0.4 μm pore size Transwell 24-well Permeable Membrane Insert (Corning). For some experiments, recombinant mouse IL-18 (Biolegend) (100 ng/ml) or vehicle was added to co-cultures. For some experiments, recombinant mouse 4-1BB ligand (Biolegend) (100 ng/ml), mouse vitronectin (Sino Biological) (100 ng/ml), and leukotriene B4 (Cayman) (100 ng/ml), or vehicle, were added to co-cultures. For some experiments, Ultra-Leaf Purified anti-mouse OX-40 agonistic antibody (clone OX-86; Biolegend, 119429) (10 μg/ml) or control Rat IgG1, κ (clone RTK2071; Biolegend, 400431) (10 μg/ml) was added to co-cultures. For some experiments, InVivoMAB anti-mouse 4-1BB agonistic antibody (clone 3H3; BioXCell, BE0239) (10 μg/ml) or InVivoMAb rat IgG2a isotype control, anti-trinitrophenol (clone 2A3; BioXCell, BE0089) (10 μg/ml) was added to co-cultures. Co-cultures were incubated for 12h. At this time, wells were subjected to one wash of 5 mM EDTA in 1x PBS, and two additional washes of 1x PBS to remove Treg cells; following this removal, Col14-LMC in wells were lysed and analyzed for RNA (see “RNA extraction and qPCR”).

RNA extraction and qPCR

RNA extraction was done using Trizol Reagent (Thermo) for lysis, followed by chloroform-based separation and precipitation of RNA with isopropanol. cDNA was created using a qScript cDNA Synthesis Kit (Quanta). qPCR was performed on cDNA using SYBR Green qPCR Master Mix (Thermo) and a Bio-Rad CFX384 qPCR system. Primers were created for this study using Integrated DNA Technology's PrimerQuest platform, and University of California Santa Clara's In-Silico PCR platform; all primers were ordered from Integrated DNA Technology and are listed in Supplemental Methods Table 1. Analysis was done by calculating ΔΔCt values relative to housekeeping gene (*Hprt*). Normalization to controls was performed between experiments.

Supplemental Methods Table 1. qPCR primers used in this study.

Hprt-FW	TCAGTCAACGGGGGACATAAA
Hprt-RV	GGGGCTGTACTGCTTAACCAG
Lif-FW	AAACGGCCTGCATCTAAGG
Lif-RV	GCAGAACCAGCAGCAGTAA
Il6-FW	CTCTCTGCAAGAGACTTCCATC
Il6-RV	CTCCGACTTGTGAAGTGGTATAG

Supplemental Methods References

De Vooght V, et al. Oropharyngeal aspiration: an alternative route for challenging in a mouse model of chemical-induced asthma. *Toxicology*. 2009;259(1-2):84-89.

Fontenot JD, et al. Regulatory T cell lineage specification by the forkhead transcription factor foxp3. *Immunity*. 2005;22(3):329-341.

Gu Z, et al. Complex heatmaps reveal patterns and correlations in multidimensional genomic data. *Bioinformatics*. 2016;32(18):2847-2849.

Han X, et al. Mapping the Mouse Cell Atlas by Microwell-Seq. *Cell*. 2018;172(5):1091-1107.e17.

La Ferlita A, et al. RNAdetector: a free user-friendly stand-alone and cloud-based system for RNA-Seq data analysis. *BMC Bioinformatics*. 2021;22(1):298.

Lex, A., et al.. UpSet: Visualization of Intersecting Sets. *IEEE Trans Vis Comput Graph*, 2014;20(12):1983-1992.

Love MI, et al. Moderated estimation of fold change and dispersion for RNA-seq data with DESeq2. *Genome Biol*. 2014;15(12):550.

Massenet-Regad L and Soumelis V. ICELLNET v2: a versatile method for cell-cell communication analysis from human transcriptomic data. *Bioinformatics*. 2024;40(3):btae089.

Nazarov, V., et al.. immunarch: Bioinformatics Analysis of T-Cell and B-Cell Immune Repertoires. 2023;GitHub.

Noël F, et al. Dissection of intercellular communication using the transcriptome-based framework ICELLNET. *Nat Commun*. 2021;12(1):1089.
Orlando F, et al. Induction of Mouse Lung Injury by Endotracheal Injection of Bleomycin. *J Vis Exp*. 2019;(146).
Sethna Z, et al. OLGA: fast computation of generation probabilities of B- and T-cell receptor amino acid sequences and motifs. *Bioinformatics*. 2019;35(17):2974-2981.
Stuart T, et al. Comprehensive Integration of Single-Cell Data. *Cell*. 2019;177(7):1888-1902.e21.
Subramanian A, et al. Gene set enrichment analysis: a knowledge-based approach for interpreting genome-wide expression profiles. *Proc Natl Acad Sci USA*. 2005;102(43):15545-15550.
